# Disentangling Effects of Vegetation Structure and Physiology on Land–Atmosphere Coupling

**DOI:** 10.1111/gcb.70035

**Published:** 2025-01-22

**Authors:** Wantong Li, Mirco Migliavacca, Diego G. Miralles, Markus Reichstein, William R. L. Anderegg, Hui Yang, René Orth

**Affiliations:** ^1^ Department of Biogeochemical Integration Max Planck Institute for Biogeochemistry Jena Germany; ^2^ European Commission Joint Research Centre (JRC) Ispra Varese Italy; ^3^ Hydro‐Climate Extremes Lab (H‐CEL), Faculty of Bioscience Engineering Ghent University Ghent Belgium; ^4^ Integrative Center for Biodiversity Research (iDIV) Leipzig Germany; ^5^ School of Biological Sciences University of Utah Salt Lake City Utah USA; ^6^ Wilkes Center for Climate Science and Policy University of Utah Salt Lake City Utah USA; ^7^ Department of Ecology Peking University Beijing China; ^8^ Faculty of Environment and Natural Resources University of Freiburg Freiburg Germany

**Keywords:** land‐atmosphere coupling, soil moisture feedback, terrestrial vegetation, vegetation physiology, vegetation structure

## Abstract

Terrestrial vegetation is a key component of the Earth system, regulating the exchange of carbon, water, and energy between land and atmosphere. Vegetation affects soil moisture dynamics by absorbing and transpiring soil water, thus modulating land–atmosphere interactions. Moreover, changes in vegetation structure (e.g., leaf area index) and physiology (e.g., stomatal regulation), due to climate change and forest management, also influence land–atmosphere interactions. However, the relative roles of vegetation structure and physiology in modulating land–atmosphere interactions are not well understood globally. Here, we investigate the contributions of vegetation structure and physiology to the coupling between soil moisture (SM) and vapor pressure deficit (VPD) while also considering the contributions of influential hydro‐meteorological variables. We focus on periods when SM is below normal in the growing season to explicitly study the regulation of vegetation on SM–VPD coupling during soil dryness. We use an explainable machine learning approach to quantify and study the sensitivity of SM–VPD coupling to vegetation variables. We find that vegetation structure and physiology exert strong control on SM–VPD coupling in cold and temperate regions in the Northern Hemisphere. Vegetation structure and physiology show similar and predominant negative sensitivity on SM–VPD coupling, with increases of vegetation dynamics leading to stronger negative SM–VPD coupling. Our analysis based on Earth system model simulations reveals that models largely reproduce the effect of vegetation physiology on SM–VPD coupling, but they misrepresent the role of vegetation structure. This way, our results guide model development and highlight that the deeper understanding of the roles of vegetation structure and physiology serves as a prerequisite to more accurate projections of future climate and ecosystems.

## Graphical abstract

This study investigates the regulation of vegetation structure and physiology in land‐atmosphere interactions. Using an explainable machine learning approach, we quantify the importance of leaf area index and transpiration on controlling the coupling between soil moisture and vapor pressure deficit during soil dryness, which are dominant in cold and temperate regions of the Northern Hemisphere. While observations show that increases in structure and physiology can lead to stronger coupling and hence potential drought propagation, Earth system models (ESMs) misrepresent structural effects. This calls for attention to more accurate representation of the respective processes in ESMs.

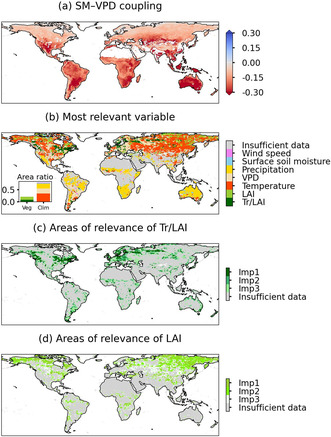

## 1 Introduction

Terrestrial vegetation is crucial in regulating the exchange of carbon, water, and energy in land–atmosphere interactions (Monteith and Unsworth 1990; Nemani et al. 2003). Thereby, vegetation translates and transports soil moisture variations to affect the atmosphere. Soil moisture (SM) availability affects vegetation functioning such that plant transpiration and albedo can change accordingly. SM also affects near‐surface temperature through evaporative cooling and the variation of solar radiation, and moreover precipitation through the modulation of moisture input to the atmosphere (Seneviratne et al. 2010). This way, SM deficits can lead to increased vapor pressure deficit (VPD) in subsequent days, with a strong negative coupling suggesting dryness propagation and amplification to the atmosphere (Van Loon 2013; Anderegg et al. 2019; Zhou et al. 2019). Thus, the correlation between SM and VPD is a useful proxy for land–atmosphere interactions. The increased covariance between SM and VPD, observed both historically and in future projections, may increase the frequency and intensity of extreme dry events and enhance drought propagation (Shekhar et al. 2024; Schumacher et al. 2022).

There are three common approaches to quantify land–atmosphere interactions: (i) the coupling between SM and surface fluxes (or atmospheric states), typically calculated using covariance or correlation (Green et al. 2017; Dirmeyer 2011; Anderegg et al. 2019; Miralles et al. 2012); (ii) controlling SM variability in Earth system models to isolate its influence on the atmosphere from internal variability or other land and oceanic forcings (Koster et al. 2004; Humphrey et al. 2021; Seneviratne et al. 2006); and (iii) determining the (de)coupling factor by evaluating the sensitivity of transpiration to stomatal conductance using site measurements (De Kauwe et al. 2017). In this study, we employ SM–VPD coupling, which is closely related to the first approach. Understanding the magnitude and drivers of SM–VPD coupling is critical, as it influences ecosystem carbon uptake, water recycling, and plant stress conditions (Novick et al. 2016; Humphrey et al. 2021). SM–VPD coupling is shaped by weather and vegetation, both spatially and temporally. Instantaneous weather conditions determine advection which acts to decouple SM and VPD as the latter is strongly affected by upstream weather conditions. Previous studies have highlighted the role of vegetation in modulating this coupling by examining spatial variations in plant functional traits and coupling strength (Anderegg et al. 2019). However, the role of physiological and structural changes in vegetation in regulating the coupling across time remains understudied (Li et al. 2024). Vegetation regulates SM–VPD coupling both physiologically, through hydraulic regulation, and structurally, through canopy development. Studying only the spatial co‐occurrence between vegetation traits and SM‐VPD coupling might underestimate the role of vegetation changes and plant water uptake strategies in moderating SM–VPD coupling towards drought propagation. The potential strong spatial relationships between canopy density and SM–VPD coupling could easily be confounded by background climate. Physiological regulation is relatively straightforward, while structural regulation involves multiple processes, as it amplifies the magnitude of evaporation and alters conditions of the land surface and atmospheric boundaries. For instance, canopy conductance and leaf area index (LAI) affect terrestrial evaporation, or “evapotranspiration” (hereafter ET), driving variations in sensible heat and VPD during drought or heatwaves (De Kauwe et al. 2017). Changes in LAI also alter biophysical properties, such as albedo and aerodynamic conductance, regulates the relationship between SM and VPD and has a potential to mitigate water or heat stress (Forzieri et al. 2020). Therefore, better understanding the regulating mechanisms of temporal vegetation dynamics on SM–VPD coupling during soil dryness periods can inform our ability to predict drought intensification over time.

Accurately representing SM–VPD coupling in Earth System Models (ESMs) is essential for predicting the likelihood of hot and dry extremes. However, modelling the role of vegetation in SM feedback is challenging due to the complexity of the biophysical and biogeochemical processes that are involved, and thus the canopy properties responsible for SM–VPD coupling in models can easily be oversimplified (Arora 2002). It is important to understand the impact of vegetation physiology and structure separately on SM–VPD coupling, as stomatal conductance and vegetation canopy structure are following different trends under historical and future climate change (Betts et al. 1997). Globally upscaled ET products and satellite‐based LAI provide valuable opportunities to understand the mechanisms through which vegetation physiology and structure regulate SM–VPD coupling (Nelson et al. 2024, preprint; Martens et al. 2017; Mu, Zhao, and Running 2013; Yan et al. 2016). Observation‐based analyses also facilitate evaluating and understanding how SM–VPD coupling is represented in ESMs. This comparison helps to assess the accuracy and reliability of ESMs in capturing the effects of vegetation on SM–VPD coupling.

In this study, we aim to identify drivers of SM–VPD coupling and quantify the sensitivity of SM–VPD coupling to changes in vegetation physiological and structural variables. To lessen the influence of external mechanisms such as sea surface temperatures that lead to inter‐annual or seasonal variability of SM–VPD coupling, we apply anomalies of SM and VPD in the correlation analysis. Given the availability of data at a global scale, we focus primarily on LAI as a key structural variable that provides information on greenness, leaf density, and vegetation cover. We also use transpiration divided by LAI (hereafter “normalised transpiration”) as a key physiological variable, as it can provide insights into canopy conductance at large scales (Zhou et al. 2023). We aim to address three overarching scientific questions: (1) Are vegetation‐related variables relevant for temporal changes in SM–VPD coupling compared to main influential hydro‐meteorological variables? (2) In which ecosystems SM–VPD coupling is dominated by vegetation and what is the sensitivity of this coupling to different vegetation variables? (3) What are the potential factors, for example, deep water resources or other properties of vegetation ecosystems, that regulate the influence of vegetation on SM–VPD coupling across space? By performing an explainable machine learning analysis, we quantify the influence of vegetation on SM–VPD coupling and disentangle specific structural and physiological influence from other potential factors (Li et al. 2021; Li et al. 2023). We employ multiple observation‐based vegetation products, from 2003 to 2020, and benchmark nine ESMs on their ability to reproduce the observational findings. To clarify the mechanisms behind the spatial patterns of coupling sensitivity to vegetation, we empirically examine the relationships between sensitivity values and properties of vegetation ecosystems (i.e., biodiversity and root‐zone water storage).

## 2 Materials and Methods

### 2.1 Data

#### 2.1.1 Observation‐Based Data

SM and VPD are derived from the ERA5‐Land reanalysis dataset (Muñoz‐Sabater et al. 2021). 1‐m SM is aggregated from 3 layers of SM and averaged by weights depending on layer depths: 7, 21, and 72 cm, respectively. VPD is calculated by air temperature and dew point temperature (Buck 1981). To test the robustness of our results to the choice of both SM and VPD from ERA5‐Land that may lead to potential inter‐dependencies or uncertainties from ERA5‐Land data assimilation, we use an in situ data‐driven and machine‐learning‐upscaled SM dataset, SoMo.ml, to replace ERA5‐Land SM (Orth 2021), and use ERA5‐Land 2‐m air temperature (hereafter “temperature”) to replace VPD and repeat the main analyses. SoMo.ml provides three layers of SM up to 50 cm deep, which are then averaged weighed by layer depth (0–10, 10–30, and 30–50 cm, respectively).

To identify the main drivers of SM–VPD coupling, we consider a suite of vegetation and hydro‐meteorological variables which have direct physical linkages with the coupling: temperature at 2 m, precipitation, VPD, wind speed at 10 m, and near‐surface SM as hydro‐meteorological variables, and normalised transpiration, and LAI, as vegetation‐related variables. Note that we do not include deep soil water as a potential main driver, since deep soil water could potentially impact on the coupling through the dynamics of vegetation‐related variables as hence it is only an indirect driver, and data of deep soil water are very uncertain and may often miss root access to groundwater. Considering that absolute values of these vegetation and hydro‐meteorological variables are easier to interpret in terms of potential biophysical mechanisms that affect SM–VPD coupling, we choose to investigate their absolute values with the coupling rather than anomalies of these vegetation and hydro‐meteorological variables.

Hydro‐meteorological data come from ERA5‐Land and LAI from the MCD15A2H Version 6 Moderate Resolution Imaging Spectroradiometer (MODIS) Level 4 (Mu, Zhao, and Running 2013). We use FLUXCOM‐X transpiration, based on an extended data‐driven framework which comprehensively integrates eddy covariance collection and other sources of Earth observations (Nelson et al. 2024, preprint). Global transpiration data are modelled and upscaled using eddy covariance‐measured ET and are validated against site‐measured sap fluxes which could have potential limitations in terms of the extrapolation ability of machine learning models and potential bias due to unclosed energy budget in eddy covariance measurements. Therefore, we also test other transpiration or ET datasets. We complement main analyses of using FLUXCOM‐X transpiration by the following variables and datasets: FLUXCOM‐X ET, GLEAM v3.6b transpiration and ET (Martens et al. 2017), and MOD16A2GF MODIS ET (Mu, Zhao, and Running 2013). We also use day‐time and night‐time microwave X‐band Vegetation Optical Depth (VOD) data from the Land Parameter Data Record (LPDR) (Du et al. 2017), which represent canopy water content. Both day‐time and night‐time VOD can be significantly affected by structural variations. We calculate monthly means of the ratio between midday and midnight VOD observations (hereafter “VOD ratio”). This ratio helps mitigate the influence of structural variations and canopy biomass on VOD, more explicitly reflecting variations related to vegetation hydraulics (Li et al. 2023; Zhang et al. 2019; Konings and Gentine 2017). However, caution is warranted, as while the VOD ratio is primarily associated with vegetation hydraulics, it can sometimes be confounded by changes in dew or temperature on leaf surfaces at the ecosystem scale (Zhao et al. 2024).

As the common period to all the observation‐based datasets is from 2003 to 2020, all data are aggregated to 0.5° spatial resolution and monthly temporal resolution covering a common 2003–2020 period. Our study area comprises regions where vegetation fractional cover is greater than 5% (Song et al. 2018) and irrigation covers less than 10% of the area (Siebert et al. 2015) to reduce impacts of human management.

#### 2.1.2 Earth System Model Data

We use publicly available data from nine models from the Coupled Model Intercomparison Project Phase 6 (CMIP6; Eyring et al. 2016) for which all considered variables are available. The information about these models is summarized in Table 1. For each model, we download daily total SM and calculate VPD based on downloaded temperature and relative humidity from CMIP6 models. We calculate daily SM–VPD coupling and aggregate it into monthly variations. We select the same hydro‐meteorological and vegetation variables (LAI, normalised transpiration, precipitation, temperature, near‐surface wind speed) at the monthly scale. We note that instead of accounting for surface SM as candidate hydro‐meteorological drivers of the coupling, we use total SM as only four out of nine models simulate surface SM. The spatial resolution of the model data is 2° such that grid cells for random forest training are significantly larger than those from the observation‐based data. We use data from the historical period from 2003 to 2014, and we test observation‐based analysis where main results are largely held in the first 12 years (see Section 3.3).

**TABLE 1:** Information about the selected earth system models.

Model name	Member	Institution	Dynamic vegetation	References
AWI‐ESM‐1‐1‐LR	r1i1p1f1	Alfred Wegener Institute, Helmholtz Centre for Polar and Marine Research	Yes	Shi et al. (2020), Lohmann et al. (2020)
IPSL‐CM6A‐LR	r1i1p1f1	Institut Pierre‐Simon Laplace	Yes	Bonnet et al. (2021)
CMCC‐CM2‐HR4	r1i1p1f1	Fondazione Centro EuroMediterraneo sui Cambiamenti	No	Scoccimarro et al. (2022)
CNRM‐CM6‐1	r1i1p1f2	Centre National de Recherches Météorologiques	No	Voldoire et al. (2019)
CNRM‐CM6‐1‐HR	r1i1p1f2	Centre National de Recherches Météorologiques	No	Voldoire and Aurore (2019)
GFDL‐CM4	r1i1p1f1	National oceanic and Atmospheric Administration, Geophysical Fluid Dynamics Laboratory	Yes	Held et al. (2019)
CNRM‐ESM2‐1	r1i1p1f2	Centre National de Recherches Meteorologiques	Yes	Seferian (2018)
UKESM1‐0‐LL	r2i1p1f2	Met Office Hadley Centre	Yes	Good et al. (2019)
CMCC‐CM2‐SR5	r1i1p1f1	Fondazione Centro EuroMediterraneo sui Cambiamenti	Yes	Cherchi et al. (2019)

### 2.2 Methods

#### 2.2.1 Quantification of SM–VPD Coupling

Land–atmosphere interactions encompass the coupling between SM and VPD. To minimize potential external influences, such as the solar cycle and long‐term global warming, we remove long‐term trends and seasonality from the daily SM and VPD data before calculating their coupling strength (Anderegg et al. 2019). The long‐term trends and seasonality are removed by subtracting the average for each month of the year and by subtracting trends constructed by a locally weighted scatterplot smoothing (LOWESS) filter. We set a smoothing parameter of 0.4 in the LOWESS filter to account for the common non‐linear trends in the long‐term variability of soil moisture or VPD that are caused by external factors. Considering the process of SM feeding back to atmospheric dryness, we quantify the coupling between SM and time‐lagged VPD. Our primary focus is to understand SM–VPD coupling, particularly the direct feedback from soil moisture and the impact of vegetation structure and physiology on the propagation of soil dryness. Examining vegetation's role in regulating soil moisture feedback during dry periods can be complicated by immediate weather variations, such as increased atmospheric demand (Novick et al. 2016). Lagged correlation helps simplify multiple mechanisms and reduce confounding effects. We tested different lag periods to calculate the coupling using correlation, and we present the main results with a 7‐day lag. Results for 1‐day and 14‐day lags are included in the Supporting Information which can also be used to test if potential remote hydro‐meteorological effects at the specific lag duration may influence the main findings. The correlation for each day is computed within a moving window surrounding the respective day; the length of this window is assigned to 30 days (Anderegg et al. 2019). By applying partial correlation and controlling for VPD at previous times, we minimize the temporal autocorrelation of VPD, allowing us to better isolate the effect of SM on subsequent variations in VPD.

The partial correlation, ${\mathrm{\rho SM}}_{t\_\mathrm{lag}},{\mathrm{VPD}}_{t}∙{\mathrm{VPD}}_{t\_\mathrm{lag}}$, can be computed using the following equation:

$$\begin{array}{c} {\mathrm{\rho SM}}_{t\_\mathrm{lag}},{\mathrm{VPD}}_{t}∙{\mathrm{VPD}}_{t\_\mathrm{lag}}=\\ \frac{{\mathrm{\rho SM}}_{t\_\mathrm{lag}},{\mathrm{VPD}}_{t}-{\mathrm{\rho SM}}_{t\_\mathrm{lag}},{\mathrm{VPD}}_{t\_\mathrm{lag}}∙{\text{ρVPD}}_{t},{\mathrm{VPD}}_{t\_\mathrm{lag}}}{\sqrt{\left(1-\rho^{2}{\mathrm{SM}}_{t\_\mathrm{lag}},{\mathrm{VPD}}_{t\_\mathrm{lag}}\right)\left(1-\rho^{2}{\mathrm{VPD}}_{t},{\mathrm{VPD}}_{t\_\mathrm{lag}}\right)}} \end{array}$$

Where ${\mathrm{\rho SM}}_{t\_\mathrm{ag}},{\mathrm{VPD}}_{t}$ is the Pearson correlation between ${\mathrm{SM}}_{t\_\mathrm{lag}}$ and ${\mathrm{VPD}}_{t}$, ${\mathrm{\rho SM}}_{t\_\mathrm{lag}},{\mathrm{VPD}}_{t\_\mathrm{lag}}$ is the Pearson correlation between ${\mathrm{SM}}_{t\_\mathrm{lag}}$ and ${\mathrm{VPD}}_{t\_\mathrm{lag}}$, and ${\text{ρVPD}}_{t},{\mathrm{VPD}}_{t\_\mathrm{lag}}$ is the Pearson correlation between ${\mathrm{VPD}}_{t}$ and ${\mathrm{VPD}}_{t\_\mathrm{lag}}$. The partial correlation is computed for *t*_lag = 1, 7, and 14 days.

**FIGURE 1:**
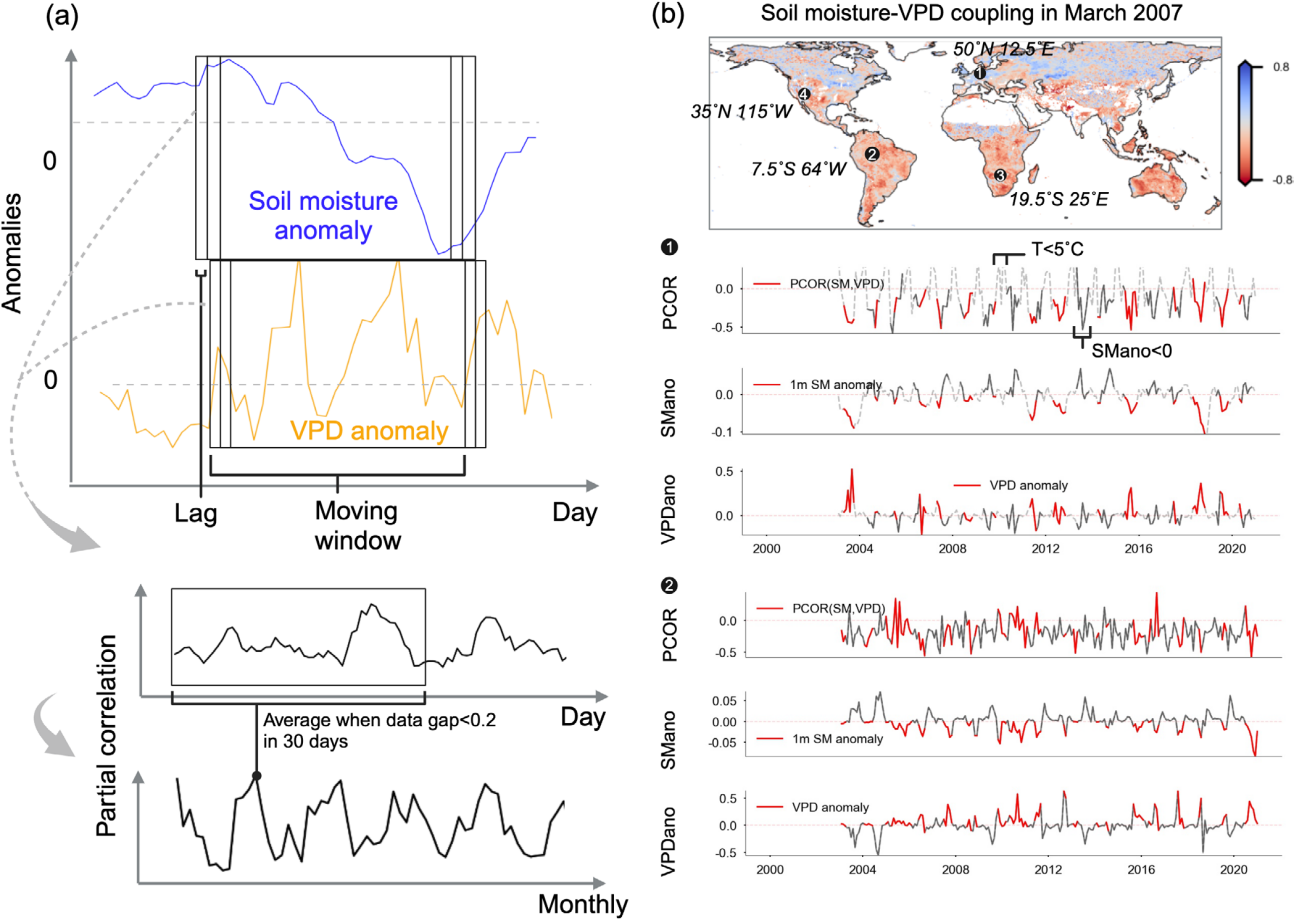
Land–atmosphere coupling as represented by the correlation between SM and lagged vapor pressure deficit (SM–VPD coupling). (a) The flowchart of SM–VPD coupling calculation and monthly result aggregation. (b) Examples of spatial and temporal variations of SM–VPD coupling of grid cells 1 and 2 (see temporal results of grid cells 3 and 4 in Figure S1). We aggregate monthly SM–VPD coupling if there are less than 20% missing daily values in a month, and we filter out non‐growing seasons and positive SM anomaly (see time series plot in b). The solid darker black line denotes periods of negative SM anomaly, the grey line denotes positive SM anomaly, and the dashed grey line denotes temperature smaller than 5°C. PCOR, partial correlation; SMano, SM anomaly; VPDano, VPD anomaly.

We aggregate SM–VPD coupling to a monthly time scale, where all the data are available, and further investigate the potential influence of vegetation and hydro‐meteorological factors. We disregard data that are related to vegetation inactive periods or positive SM anomalies. We focus on periods with negative SM anomalies as land–atmosphere coupling is typically stronger and more relevant during periods of drought stress. Non‐growing periods are defined as times when monthly temperature falls below 5°C. We also test a definition based on the temperature threshold plus gross primary productivity being greater than 0 in the supplementary. The procedure of calculating SM–VPD coupling is illustrated in Figure 1, and the spatial and temporal variations of the coupling are presented with examples. Strongly negative correlation coefficients can be found in semi‐arid and arid regions.

#### 2.2.2 Determining the Drivers of SM–VPD Coupling

We conduct an analysis using an explainable machine learning approach to identify the main influential factors of SM–VPD coupling. Specifically, we train a random forest model for each grid cell to predict SM–VPD coupling at the monthly scale (see Section 2.1.1), using the absolute values of all considered vegetation and hydro‐meteorological variables as they are capable of interpreting physical links (see variable list in Section 2.1.1). Random forests are chosen because they do not require prior assumptions about data distribution and can handle data with non‐linear relationships (Breiman 2001). The hyperparameter settings for the random forest models are as follows: the number of estimators is set to 100, the maximum features parameter is set to 0.3, bootstrap sampling is enabled, and the random state is set to 42.

Random forest models allow for random splitting of data into training and testing samples with bootstrap sampling strategies. The performance of each random forest model is evaluated by computing the *R* ^2^ between the observed target variable and the modeled results using testing data samples not employed in the model training, referred to as “out‐of‐bag *R* ^2^” (Breiman 2001). To enhance the robustness of each model, we incorporate data from neighboring grid cells, thereby increasing the sample size while maintaining data homogeneity. Specifically, a 3 × 3 grid cell matrix is used to train the model for the core grid cell, as neighboring grid cells typically exhibit similar climate and vegetation conditions. We exclude grid cells with fewer than 20 data samples from the analysis.

Note that regions with out‐of‐bag *R* ^2^ less than 0.2 are filtered out. Areas with sparse vegetation or dense irrigation are also excluded. This ensures that the random forest model's performance in the remaining regions is suitable for interpreting the results. For the remaining grid cells, we calculate SHapley Additive exPlanations (SHAP) values to (i) identify the importance of each variable and (ii) infer the physical driving mechanisms for the vegetation variables of interest while disentangling the contributions of hydro‐meteorological variables. Note that the SHAP analysis helps disentangle the marginal contributions of different variables to the predictions by accounting for interactions between predictor and target data, both locally and globally (Lundberg and Lee 2017). To evaluate ESMs, we perform the same analysis by applying random forests and SHAP values on model outputs, and compare the sensitivity of SM–VPD coupling to vegetation structure and physiology between models and observations.

To determine variable importance, we calculate the absolute values of the SHAP marginal contributions for each predictor and rank them accordingly. As SHAP values calculated based on random forest predictions cannot be compared between different random forest models, we identify the relatively influential predictor variables based on their ranking. To infer the influence of structure (i.e., LAI) and physiology (i.e., normalised transpiration) on SM–VPD coupling, we fit Theil‐sen regression models on SHAP values and LAI, or on SHAP values and normalized transpiration, respectively (Gilbert 1987). The regression slope then represents the sensitivity of SM–VPD coupling to LAI or normalized transpiration. Theil‐sen slopes are relatively more robust against outliers compared to ordinary least squares regression.

### 2.3 Auxiliary Data and Spatial Analysis

After determining the influence of LAI and normalized transpiration on SM–VPD coupling in each grid cell, we aim to understand the resulting spatial patterns across the globe. To achieve this, we average the importance of each variable across different climates, as defined by the Köppen–Geiger classification (Linscheid et al. 2021; Beck et al. 2023). Additionally, we calculate the long‐term mean aridity and temperature using ERA5‐Land, with aridity defined as the ratio between net radiation (converted into mm) and precipitation (Budyko and Miller 1974). We summarize the sensitivity values of SM–VPD coupling to different variables by representing the median sensitivity values across different Köppen–Geiger climate zones and across gradients of aridity and temperature. This analysis allows us to capture and illustrate how the influence of LAI and normalized transpiration on SM–VPD coupling varies spatially under different climatic conditions.

To further enhance our understanding of ecosystem conditions in altering the impact of LAI and normalized transpiration on variations of SM–VPD coupling, we incorporate additional variables, species richness of native plants (Ellis, Antill, and Kreft 2012; Kreft and Jetz 2007) and root‐zone water storage capacity (Stocker 2021). These variables describe spatial heterogeneity of vegetation ecosystems and may affect ecosystem regulation on SM–VPD coupling. We calculate partial correlations between plant biodiversity or water storage capacity and the coupling sensitivity results across different regions, controlling for aridity and temperature, to better understand additional functions of biodiversity and water storage beyond background climate variability. Recognizing that plant biodiversity and root‐zone water storage capacity may have regional rather than global effects on sensitivity values, we compute correlations within each class of temperature and aridity instead of across the entire study area at once. However, testing correlations across multiple regions and variables poses a risk of multiple testing issues, where the likelihood of false positives increases due to the numerous statistical tests conducted simultaneously. To address this, we account for multiple testing and limit the proportion of falsely rejected null hypotheses. This allows us to better assess the overall effect of plant biodiversity or root‐zone water storage capacity on SM–VPD coupling sensitivity (Cortés et al. 2020). Specifically, we employ the Benjamini–Hochberg procedure to adjust two‐sided p‐values, thereby controlling the false discovery rate across grid cells and the two variables (Benjamini and Hochberg 1995).

## 3 Results and Discussion

### 3.1 Drivers of SM–VPD Coupling

**FIGURE 2:**
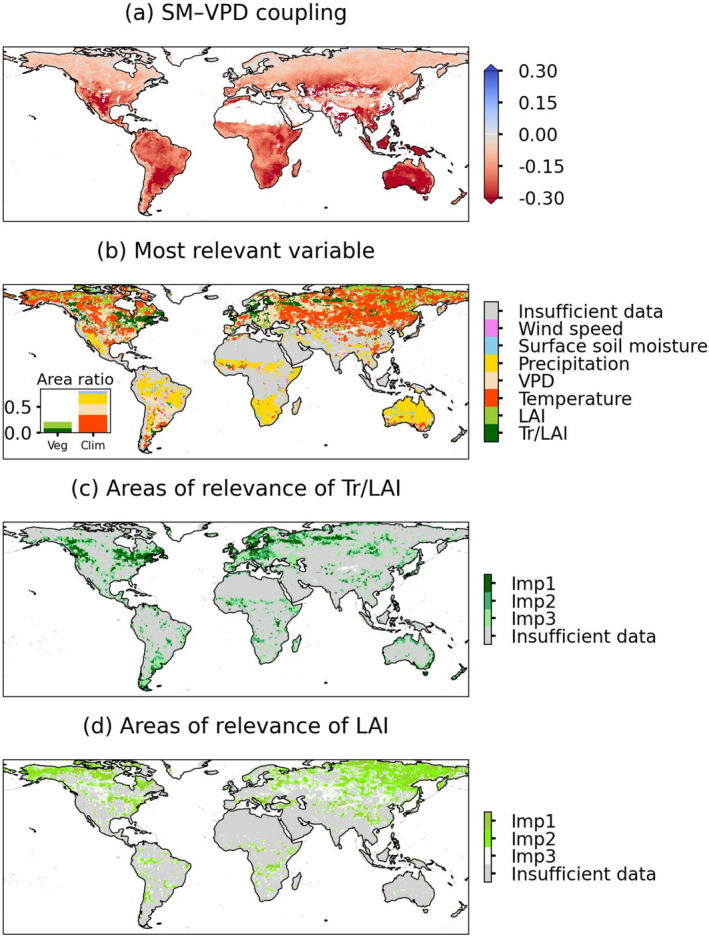
Main vegetation and hydro‐meteorological factors influencing SM–VPD coupling. (a) Distribution of averaged SM–VPD coupling during 2003–2020. (b) Spatial distribution of variables that are identified as the main drivers based on SHAP analysis. The inset shows ratios of land area where each variable is the most important factor; Veg, vegetation variables; Clim, hydro‐meteorological variables. The order of the legend in (b) is random while the color on the global map indicates the most important variable with each grid cell one variable. (c) Mapping of regions where the physiological variable, normalized transpiration (Tr/LAI), is identified as the first (Imp 1), second (Imp 2), or third most influential variable (Imp 3). (d) Like (c) but for LAI. Map lines delineate study areas and do not necessarily depict accepted national boundaries.

After we filter out non‐growing seasons and positive SM–VPD correlation to study regions with strong coupling regimes, we quantify the sensitivity values of SM–VPD coupling to its vegetation and hydro‐meteorological drivers. We find strong negative coupling in semi‐arid and arid regions in Figure 2a such as central North America, Sahel, central Eurasia, South Africa, and Australia, with exceptions in tropical Asia, in line with previous studies investigating land–atmosphere coupling (Dirmeyer 2011; Miralles et al. 2012). Figure 2b illustrates the spatial distribution of the most important variables of SM–VPD coupling for which we rank the variable importance and map the first important one for each grid cell. About 25% of the study area SM–VPD coupling seems to be mostly influenced by vegetation (LAI and normalised transpiration), while for 75% of the study area climate shows the major influence. The influence of vegetation is consistent with the results from previous studies that quantify the variance explained by plant hydraulic and photosynthetic traits (Anderegg et al. 2019). In addition, in Figure S2 we map the spatial distribution of the second and the third most important variabels in our study area. Compared to results in Figure 2b, more grid cells show that normalised transpiration is selected as the second most important variable and LAI is selected as the third most important variable. The ratio of variable importance values between the second and the first most important variable is greater than 0.8 over half of the global results, suggesting similar relevance of the second‐order drivers compared to the first‐order drivers in regulating SM–VPD coupling (Figure S2).

We further disentangle the influence of vegetation structure and physiology and map all regions where they are among the most relevant three drivers. Figure 2c,d highlights the geographical regions in the Northern Hemisphere where vegetation structure (represented by LAI) and vegetation physiology (represented by normalized transpiration) dominate the coupling. Plant physiology is identified as a main vegetation factor influencing coupling in about half of the Northern Hemisphere. This includes regions such as Europe, central Northeast America, parts of southern South America, the Sahel, and the eastern belt of Australia. Vegetation structure is relevant in about two thirds of the Northern Hemisphere, mostly northeast Eurasia, northern and central North America, and some grid cells in central and Southern South America, and South Africa.

Random forest models predict SM–VPD coupling with varying accuracy across different regions (Figure S3). Compared to tropical regions, random forest models more accurately predict SM–VPD coupling in the Northern Hemisphere, except for West Asia and South Asia. The models also perform better in southern South America, as well as in semi‐arid and arid areas of the Sahel, South Africa, and Australia. The limited predictive ability of the model in the tropics, West Asia, and South Asia may be due to relatively sparse observations used in the employed remote‐sensing datasets caused by frequent cloud cover and high vegetation density in tropical regions, uncertainties in hydro‐meteorological reanalysis data, and intensive human activities such as harvesting in agricultural areas (Li et al. 2021).

### 3.2 Sensitivity of SM–VPD Coupling to Its Drivers

**FIGURE 3:**
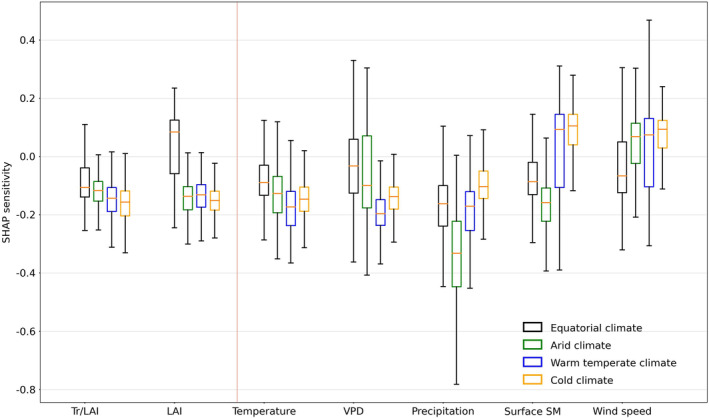
SHAP sensitivity of SM–VPD coupling to vegetation and hydro‐meteorological variables across climates.

In Figure 3, we summarize the sensitivity of the considered predictors for SM–VPD coupling across climates from the Köppen–Geiger classification (Figure S4). Overall, negative sensitivities of the coupling can be found in most considered variables, indicating that the increase in the variable may strengthen the negative SM–VPD coupling. Precipitation and surface SM show the most negative sensitivity in arid climate. A little increase of water availability in arid regions could easily strengthen the coupling between SM and VPD, which is likely due to the non‐linearity of the SM feedback (Seneviratne et al. 2010). Temperature and VPD both show the strongest negative sensitivity in warm temperate climates. This is related to relatively high VPD and temperature averages across growing seasons (Figure S5e), so that additional small increases of temperature can trigger much stronger SM–VPD coupling.

Normalised transpiration shows a widespread negative sensitivity. Increases in normalised transpiration commonly contribute to a stronger coupling due to its physical link to the latent heat when SM is available to use. However, the sensitivity of the coupling to normalised transpiration differs across climates, with the weakest negative sensitivity in equatorial climate, followed by arid, warm temperate, and cold climates. The weakest negative sensitivity in equatorial climate may be due to two reasons: additional water availability from soil layers deeper than 1 m (Mu et al. 2021) and vegetation physiology there is less controlled by biotic factors such as stomatal conductance but more controlled by abiotic factors such as light or temperature (De Kauwe et al. 2017).

LAI shows similarly negative coupling sensitivities in most climates, but the coupling in cold climates shows the most negative sensitivity whereas the coupling in equatorial climates shows overall positive sensitivity. This is related to the different roles of LAI in regulating soil moisture and latent heat flux and in altering biophysical conditions across climates. In cold regions with low to moderate vegetation cover, increases in LAI could likely reduce surface albedo and aerodynamic resistance, and increase temperature, enhancing the land–atmosphere coupling (Forzieri et al. 2017). Our results align with a previous study that examined land surface temperature sensitivity to LAI, noting strong sensitivity in middle and high latitudes but weak sensitivity in tropical regions (Chen et al. 2020; Hales, Neelin, and Zeng 2004). This low sensitivity in tropical areas is expected, as LAI often reaches saturation and does not cause significant changes in biophysical factors like aerodynamic resistance, resulting in relatively low coupling sensitivity. We illustrate the potential processes in schematic Figure S6 which illustrates the potential impact pathways of vegetation and hydro‐meteorological variables on SM–VPD coupling. The background climates in Figure S6 highlight the underlying main influential variables presented in Figure 3. Given our interest in investigating the explicit roles of vegetation structure and physiology in regulating SM–VPD coupling, we focus on understanding the role of normalised transpiration and LAI in the next sections.

### 3.3 Comparing Vegetation Regulation of SM–VPD Coupling Between Observations and Models

**FIGURE 4:**
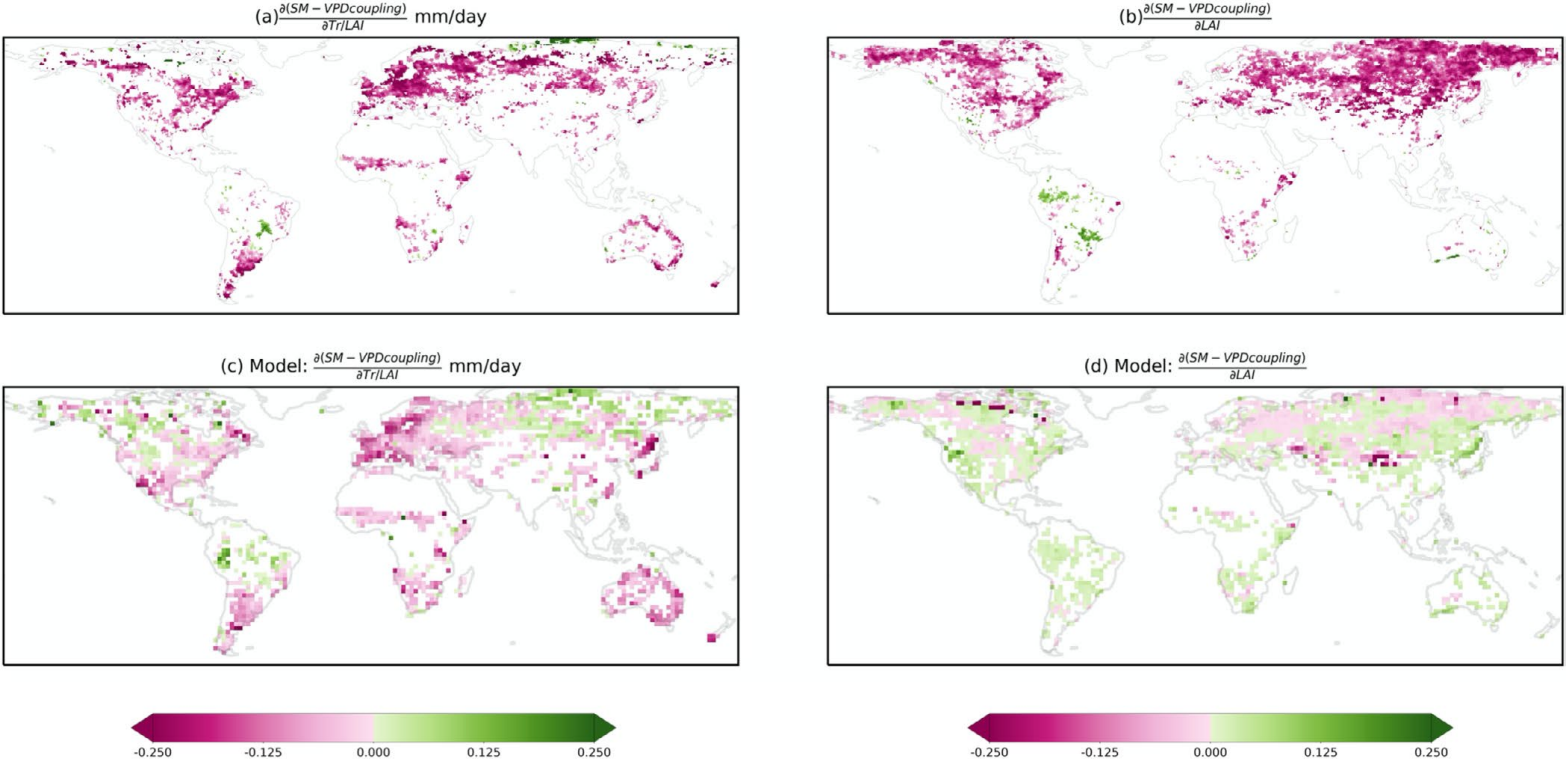
Sensitivity of SM–VPD coupling to vegetation physiology and structure from observation‐based (a, b, OBS) or Earth System Model‐based (c, d, Model) data. Results are assessed using the two‐sided significance test at the *p* < 0.01 level with Theil‐sen regressions for each grid cell. Map lines delineate study areas and do not necessarily depict accepted national boundaries.

We map the global sensitivity of SM–VPD coupling to vegetation structural and physiological variables as expressed through normalized transpiration and LAI (Figure 4). We repeat this analysis using simulations from CMIP6 ESMs. We find that, while ESMs can largely reproduce the negative sensitivity of SM–VPD coupling to normalized transpiration as observed in many regions, the simulated sensitivity of SM–VPD coupling to LAI does not match observation‐based patterns. For normalized transpiration, both observations and models show the strongest negative sensitivity in Western and Northern Europe, as well as in a few regions in Southern South America and Eastern Australia. Some regions in Siberia and South America exhibit positive sensitivity in observation‐based results, which can be reproduced by the models, although models overestimate areas with positive sensitivity. The coupling sensitivity to LAI in ESM simulations is negative as observed in some boreal and temperate areas but is largely misrepresented in the Southern Hemisphere.

A similar overestimation of LAI's impact on land–atmosphere coupling, calculated using an empirical metric representing the coupling of vegetation transpiration to stomata or aerodynamic processes, is noted in Zhang et al. (2022). While the modeled coupling between SM and VPD closely matches the distribution calculated using ERA5‐Land reanalysis data, the discrepancies in ESMs regarding SM–VPD coupling sensitivity to LAI can have various reasons (Figure S7). The model discrepancies could stem from (i) overestimation in simulated LAI in ESMs (Figure S7c), and (ii) relatively high uncertainties in random forest models when disentangling drivers of SM–VPD coupling when using modelled data with coarse spatial resolution (Figure S7e,f). Furthermore, the modelled influence of LAI and canopy structure on processes determining land–atmosphere coupling such as stomatal or aerodynamic conductance (Mallick et al. 2024) may play a role. Finally, potential biases in land initial conditions could contribute to misrepresenting the drivers of SM–VPD coupling in models (Seo et al. 2024).

We test the robustness of our findings regarding the widespread negative sensitivities of SM–VPD coupling to LAI and normalised transpiration from observation‐based results. For this purpose we use different datasets to recalculate our analyses, including Fluxcom‐X transpiration, normalized transpiration or ET, GLEAM transpiration or ET, and a MODIS ET product. The global pattern remains largely unchanged (Figure S8a–d). Vegetation physiological controls on SM–VPD coupling inferred from transpiration products are also in line with results inferred from an independent data source from microwave remote sensing, vegetation optical depth (VOD). We use VOD ratio which is the ratio between midday and midnight VOD observations to explicitly focus on the information of plant hydraulic regulation, with increased values reflecting stronger stomatal and xylem control. VOD ratio shows predominant positive sensitivity (Figure S8e), suggesting that decreases in stomatal conductance could mitigate soil drought propagation during the soil drying period. In addition to structural and physiological variables of vegetation, some emerging ecosystem properties might also be relevant for regulating SM–VPD coupling and are useful to assess considering the need for vegetation management and model development. For example, we also study water use efficiency (defined as the ratio between GPP and transpiration; Bonan et al. 2014) in regulating SM–VPD coupling (Figure S8f). In temperate and arid climates, SM–VPD coupling shows positive sensitivity to water use efficiency in Europe and southern North America. Increases in water use efficiency is linked to reductions in stomatal conductance and transpiration under soil dryness with less related reductions (or slight increases) in vegetation carbon uptake. This implies that increases in water use efficiency weaken SM–VPD coupling and mitigating the likelihood of drought propagation.

**FIGURE 5:**
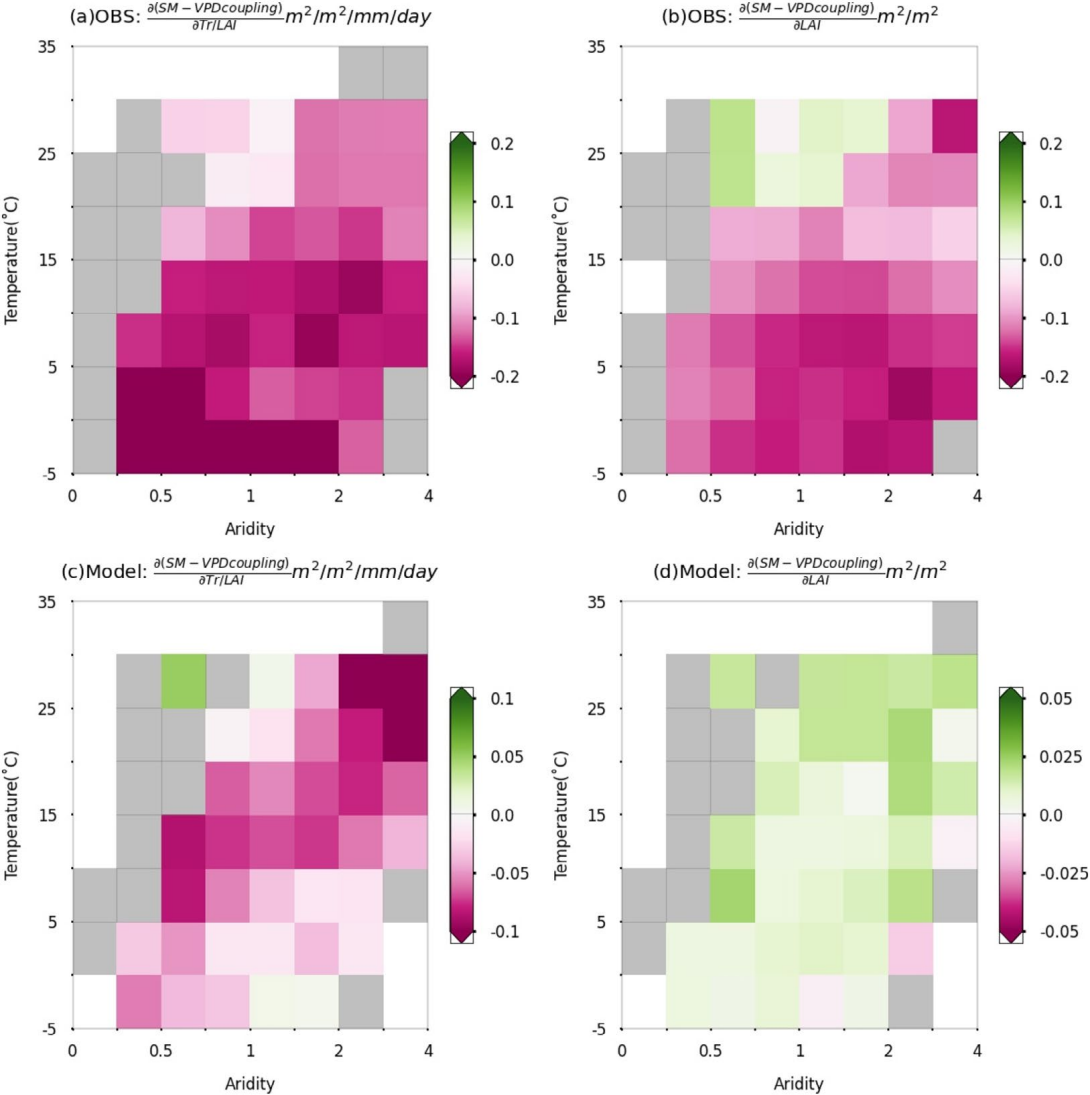
Sensitivity of SM–VPD coupling to vegetation physiology and structure across grid cells across gradients of aridity and temperature. (a, b) Observation‐based products (OBS). (c, d) ESMs (Model). Mean values of sensitivity are shown with colors in each bin, and grey denotes that bins have less than 10 grid cells with significant results.

Next, we summarize the sensitivity of SM–VPD coupling to vegetation structure and physiology across grid cells with similar temperature and aridity. We find that the observed sensitivity changes predominantly along gradients of aridity and temperature (Figure 5a,b). Wet and cold regions show the strongest negative sensitivity to normalized transpiration, while dry and cold regions show the strongest negative sensitivity to LAI. Although the overall sensitivity is largely negative for both vegetation structure and physiology, their different impacts on SM–VPD coupling are likely related to impacts of LAI on surface properties. Nevertheless, ESMs could not capture these patterns of sensitivity variations along temperature and aridity gradients. Note that individual models show divergent patterns from this average result, especially for the physiological regulation (Figure S9).

The main observation‐based patterns of sensitivity variations along temperature and aridity gradients hold under several conditions: (i) when plotting results only from grid cells where vegetation is the most or second most influential (Figure S10), (ii) when using different SM lag times in the calculation of SM–VPD coupling (Figure S11) or when replacing ERA5‐Land SM with observation‐based machine learning‐upscaled SM, SoMo.ml (see Section 2.1.1; Figure S12), and (iii) when using different definitions of growing seasons (Figure S13a,b). Vegetation structure and physiology could be correlated with each other, and such collinearity could affect our results. To test this, we perform additional analyses: (i) We only consider one of the LAI and normalised transpiration variables and re‐run the random forest analysis. The results show little changes, suggesting no major influence of potential collinearity of vegetation structure and physiology (Figure S13c,d); (ii) We also present global results when removing a few grid cells with high multi‐collinearity among vegetation variables (Figure S14); (iii) Results from VOD ratio suggest similar physiological influence on SM–VPD coupling (Figure S8e). Furthermore, Figure S13e,f demonstrates that when focusing on a shorter period, corresponding to the ESM‐based analysis (2003–2014), SM–VPD coupling sensitivity to normalized transpiration and LAI is largely similar to the main analysis based on data from 2003 to 2020.

**FIGURE 6:**
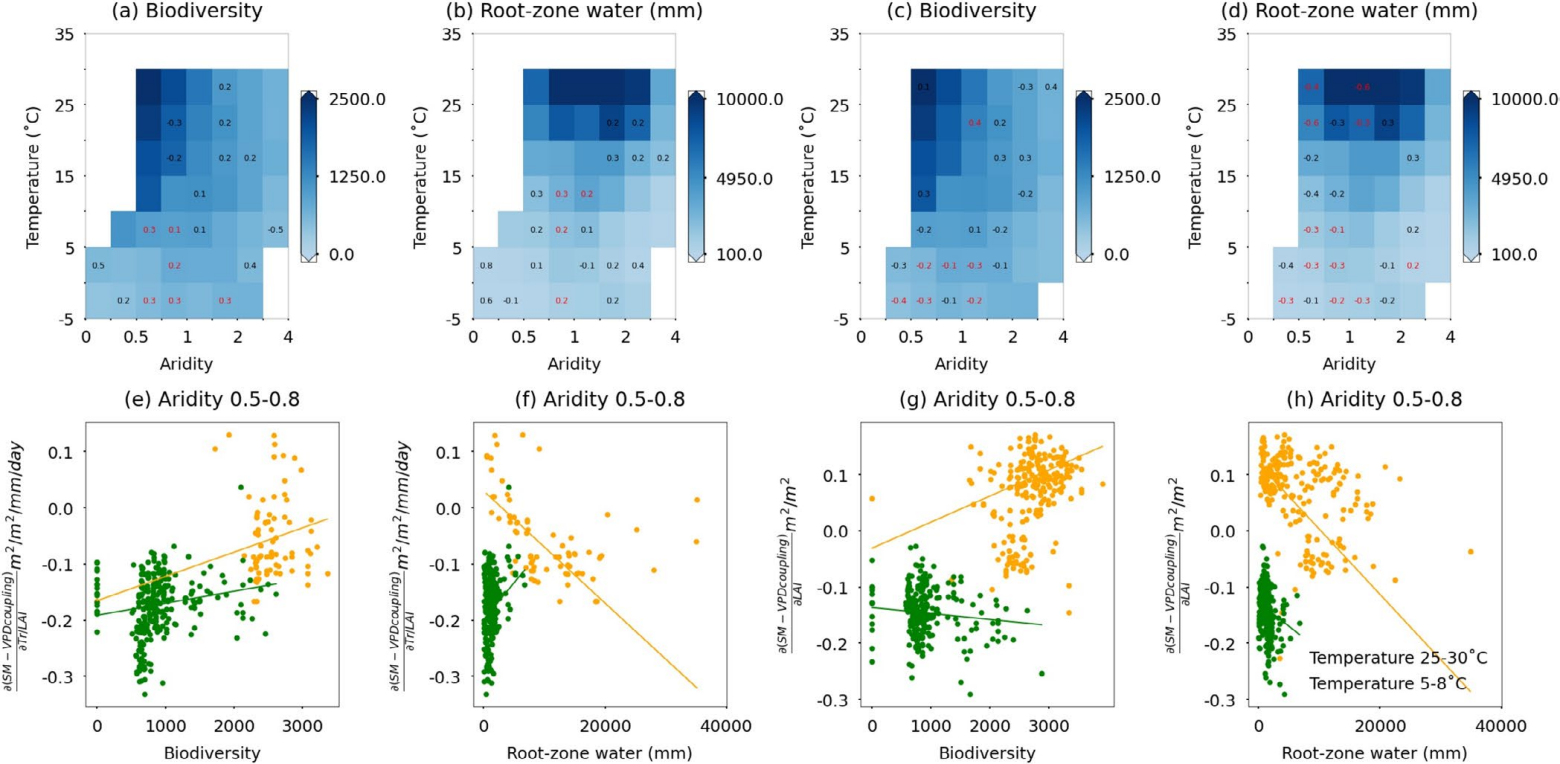
The distribution and relevance of plant biodiversity and root‐zone water storage capacity on SM–VPD coupling sensitivity to vegetation physiology and structure. (a, b) Partial correlation between biodiversity (or root‐zone water storage capacity) and SM–VPD coupling sensitivity to normalized transpiration. (c, d) The same analysis as (a, b), but focusing on SM–VPD sensitivity to LAI. (e, f) present values for biodiversity (or root‐zone water storage capacity) and SM–VPD coupling sensitivity to normalized transpiration from selected grid cells where aridity ranges from 0.5 to 0.8 and temperature is between 25°C and 30°C (orange) and 5°C–8°C (green). Linear lines are fitted using Theil‐sen regression. (g, h) Similar to (e, f), but for SM–VPD sensitivity to LAI. Biodiversity data is represented by the native richness of plants. (a–h) apply the two‐sided significance test at the *p* < 0.05 level, as assessed by partial spearman correlation for each bin, controlling for the dependence of temperature and aridity. The partial correlation coefficient is shown in black labels in each bin. Red labels additionally indicate that the results are robust to multiple tests of significance using the Benjamini–Hochberg procedure at the *p* < 0.05 level. Bins are disregarded with numbers of grid cells smaller than 20.

In the next step, we study potential relevance of water availability and biodiversity on the sensitivity of SM–VPD coupling to vegetation structure and physiology across space and present results in Figure 6. For this, we implement a partial correlation analysis which accounts for the relationships between sensitivity values and water availability (represented by root‐zone water storage capacity), or between sensitivity values and biodiversity (represented by species richness of native plants). We perform this analysis for each aridity and temperature group, because we find that relationships between biodiversity and coupling sensitivity vary significantly in specific regions (Figure 6e–h). Since sensitivity values, biodiversity, and water availability all depend on the background climate, we control for the potential dependence of sensitivity on temperature and aridity in the correlation analysis.

We find that biodiversity and water availability are mostly positively correlated with the sensitivity of SM–VPD coupling to normalized transpiration in cold regions (Figure 6a,b). This suggests that higher plant diversity or sufficient water in a grid cell makes the SM–VPD correlation less negative (or weakening SM–VPD coupling) and thus mitigate the potential of soil drought propagation (Mahecha et al. 2024; Anderegg et al. 2018). Ecosystems with high biodiversity potentially enhance water retention and saving strategies due to diverse soil and plant physiological traits as well as microclimatic conditions (Brunner et al. 2021; Beugnon et al. 2024). Therefore, both biodiversity and water availability likely contribute to increased evaporative cooling in the presence of near‐surface soil dryness. We find contrasting results for drivers of the sensitivity of the SM–VPD coupling to LAI. Biodiversity and water availability are mostly negatively correlated with the sensitivity of the coupling to LAI in cold regions and in some warm regions (Figure 6c,d). During near‐surface soil dryness, LAI can be maintained or even increased under plant‐diverse and water‐rich conditions, potentially increasing sensible heat flux and strengthening SM–VPD coupling. After applying the Benjamini–Hochberg procedure to account for potential multiple hypothesis tests, half of the aridity‐temperature subregions remains to show significant relationships.

Understanding the feedback of SM on VPD is crucial, as it can either mitigate or amplify VPD signals, impacting vegetation and the water cycle (Canadell et al. 2021; Dai, Zhao, and Chen 2018). Our study highlights the role of vegetation structure and physiology in regulating the temporal strength of SM–VPD coupling and drought intensification, which has significant implications for drought mitigation and adaptation strategies. Multiple lines of evidence suggest that increases in normalized transpiration and LAI can strengthen SM–VPD coupling across many regions (Figure 3 and Figure S8a–c). This is because both transpiration and LAI enhance latent heat, a key driver of SM–VPD coupling (Seneviratne et al. 2010). Interestingly, equatorial ecosystems exhibit the weakest influence on the intensification of SM–VPD coupling and drought propagation, while cold ecosystems show the strongest effects. Two primary factors contribute to this difference: (1) During dry periods, vegetation in equatorial regions may extract water from soil layers deeper than 1 m (Mu et al. 2021), leading to an apparent decoupling of the SM–VPD relationship based on 1 m soil moisture; (2) In equatorial climates, transpiration is primarily influenced by abiotic factors such as light and temperature, rather than biotic factors like stomatal conductance (De Kauwe et al. 2017). Thus, enhanced monitoring of ecosystem responses to atmospheric changes is necessary to understand the mechanisms of drought intensification, particularly in broad cold ecosystems. Unlike normalized transpiration and LAI, water use efficiency can weaken SM–VPD coupling in over half of global ecosystems, notably in southern North and South America and parts of northern and western Europe (Figure S8f). Considering that the same water loss but diverse carbon gain can alter albedo and aerodynamic properties, future land management strategies must consider the balance between carbon assimilation and water loss in mediating land–atmosphere coupling.

This study examines the monthly variations in SM–VPD coupling. To ensure that the sensitivity of this coupling to LAI and normalized transpiration is not influenced by factors such as regional climatology, we tested for adding months in our random forest modeling. Our main findings remain valid, as shown in Figure S13g,h. However, we acknowledge four main limitations in our analysis: (i) the selection of time scales, (ii) the identification of drivers influencing SM–VPD coupling, (iii) the inability to infer causality from the observed relationships, and (iv) the potential remote effects of soil moisture feedback. Since temporal scale complicates the study of SM–VPD coupling, the relevance of vegetation physiology and structure may change across different time scales. Vegetation physiology might become more significant than structure due to its quicker response to stress conditions (Li et al. 2023). Future research could investigate SM–VPD coupling at various temporal scales to better understand the evolving mechanisms of vegetation regulation. Another potential limitation is that including surface SM and VPD in the analysis may weaken the role of vegetation dynamics in regulating SM–VPD coupling and impose some potential circularity. We thus tested the removal of surface SM and VPD and found that global maps of coupling sensitivity to normalized transpiration and LAI remain largely unchanged (Figure S13i,j). However, model performance does not change much in most ecosystems when excluding these factors, with out‐of‐bag *R* ^2^ reductions mostly below 0.05 (Figure S15). Southeastern South America shows more significant differences, likely due to the strong feedback of surface SM on surface fluxes compared to vegetation feedback (Erfanian et al. 2022). When we examined global maps of the most relevant variables and areas related to normalized transpiration and LAI, we noted few differences between analyses with and without surface SM and VPD, except for an increased relevance of transpiration in northern Europe (Figure S16). It is important to emphasize that we use absolute values of surface SM and VPD as explanatory variables, which differ from calculating the coupling between SM and VPD anomalies. Additionally, the parameter settings in the calculation of anomalies have minimal influence on the main results (Figure S13k,l).

Since the temporal dynamics of transpiration are influenced by hydro‐meteorological variables such as radiation, temperature, and VPD, caution is warranted as normalized transpiration still has the potential to overestimate physiological regulation. To mitigate potential confounding effects of hydro‐meteorological changes on vegetation regulation, we chose to retain surface soil moisture (SM) and vapor pressure deficit (VPD) as explanatory variables in the random forests. Because potential multi‐collinearity can likely reduce the performance of explainable machine learning in disentangling physiological and structural regulation from hydro‐meteorological regulation, we also provide global results after testing for multi‐collinearity. The results for normalized transpiration are largely unchanged (Figure S14). The global result shows that some grid cells in northern latitudes are masked due to relatively high multi‐collinearity between LAI and other variables considered. In addition, we also test adding incoming shortwave radiation into random forest models to account for potential confounding effects of meteorological changes on normalised transpiration and find our main results to still hold (Figure S13m,n). However, our explainable machine learning approach does not establish causality in the physical mechanisms between vegetation and SM–VPD coupling, despite our efforts to incorporate as many predictor variables as data availability allows. In the end, we acknowledge that remote effects of soil droughts can contribute to up to 15% reductions of single rainfall events and up to 30% in rainfall reductions during individual months in drylands (Schumacher et al. 2022), while quantifying the influence of such upwind heat advection and remote SM feedback falls outside the scope of our study. Future work needs to better quantify vegetation regulation on both local and remote variations of SM–VPD coupling or other types of land–atmosphere interactions.

## 4 Conclusions

Vegetation plays a relatively important role in regulating SM–VPD coupling, explaining the temporal variability of the coupling beyond that explained by hydro‐meteorological variables alone. Structural and physiological changes in vegetation have a detectable, widespread influence on the strengthening of SM–VPD coupling across the globe, particularly in cold regions. Going beyond previous research, temporal variations in the structural and physiological influence of vegetation on SM–VPD coupling are now explicitly studied which vary between ecosystems and involve different biochemical and biophysical processes. For example, physiology affects transpiration and hence SM–VPD coupling through stomatal regulation and photosynthetic regulation, while structure affects SM–VPD coupling through for example, changes in leaf area and hence evaporative cooling. In addition, there are other land surface properties that affect land–atmosphere coupling, such as aerodynamic resistance and albedo, which are related to vegetation structure but not directly physiology. ESMs can largely reproduce the role of vegetation physiology for the SM–VPD coupling, but not for the case of vegetation structure. This seems to be due to several reasons, including some overestimation of LAI distribution and coarse spatial resolution in models.

In summary, our results provide a better understanding of the influence of vegetation on land–atmosphere coupling. This is done using a powerful data‐driven statistical approach that considers a comprehensive suite of potential drivers of the coupling, and can isolate the role of vegetation physiology and structure among them. Furthermore, our results provide important guidance for the future development of Earth system models regarding the biophysical regulation of vegetation structure on the SM feedback, which can then contribute to more accurate projections of future climate. This is particularly true for the climate that is strongly mediated by the land surface, such as soil drought and its propagation to the atmosphere. Future improvement of these models will provide new and valuable opportunities to study processes and drivers of land–atmosphere coupling from a mechanistic perspective, which will be an important complement to our statistical analyses of observational data.

## Author Contributions

**Wantong Li:** conceptualization, formal analysis, methodology, visualization, writing – original draft, writing – review and editing. **Mirco Migliavacca:** conceptualization, formal analysis, methodology, writing – review and editing. **Diego G. Miralles:** writing – review and editing. **Markus Reichstein:** writing – review and editing. **William R. L. Anderegg:** writing – review and editing. **Hui Yang:** writing – review and editing. **René Orth:** conceptualization, formal analysis, methodology, writing – review and editing.

## Conflicts of Interest

The authors declare no conflicts of interests.

## Supporting information


Figure S1.

## Data Availability

The data and code that supports the findings of this study are available from Zenodo at https://zenodo.org/records/14560928. ERA5‐Land soil moisture (SM) and vapor pressure deficit data were obtained from the Copernicus Climate Change Service (C3S) Climate Data Store at https://doi.org/10.24381/cds.e2161bac. SoMo.ml data were obtained from Figshare at https://doi.org/10.6084/m9.figshare.14790510. Transpiration data were obtained from the ICOS ERIC ‐ Carbon Portal at https://doi.org/10.18160/5NZG‐JMJE. Evapotranspiration and latent heat flux data were obtained from the NASA Land Processes Distributed Active Archive Center at https://doi.org/10.5067/MODIS/MOD16A2GF.061. Transpiration and evapotranspiration data were obtained from the Global Land Evaporation Amsterdam Model (GLEAM) at https://www.gleam.eu/ (v3.6b). The LPDR vegetation optical depth data were obtained from the NASA National Snow and Ice Data Center Distributed Active Archive Center at https://doi.org/10.5067/RF8WPYOPJKL2. CMIP6 model simulation data were obtained from the Earth System Grid Federation at https://doi.org/10.22033/ESGF/CMIP6.9328, https://doi.org/10.22033/ESGF/CMIP6.5195, https://doi.org/10.22033/ESGF/CMIP6.3823, https://doi.org/10.22033/ESGF/CMIP6.4066, https://doi.org/10.22033/ESGF/CMIP6.4067, https://doi.org/10.22033/ESGF/CMIP6.8594, 10.22033/ESGF/CMIP6.4067, 10.22033/ESGF/CMIP6.6113, and https://doi.org/10.22033/ESGF/CMIP6.3825.
