# Supplementary material for: Disentangling Effects of Vegetation Structure and Physiology on Land–Atmosphere Coupling
Source: Glob Chang Biol. 2025 Jan 22;31(1):e70035. doi: 10.1111/gcb.70035 (PMC11751765; doi:10.1111/gcb.70035)
Supplement: Supplementary file 1 — Figure S1. [file GCB-31-e70035-s001.pdf]

Supplementary materials including Figures S1-16.

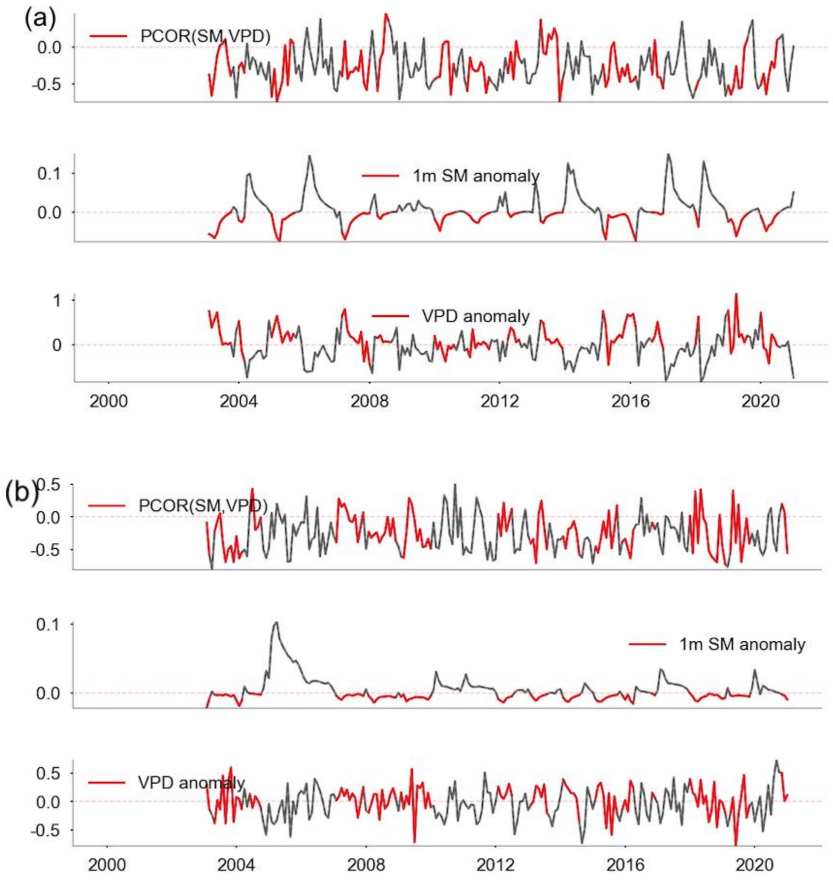

Figure S1: Temporal variations of SM–VPD coupling and anomalies of SM and VPD in examples of two grid cells. Like the time series of coupling results in Figure 1b but for grid cells 3 and 4. PCOR: partial correlation; SMano: SM anomaly; VPDano: VPD anomaly.

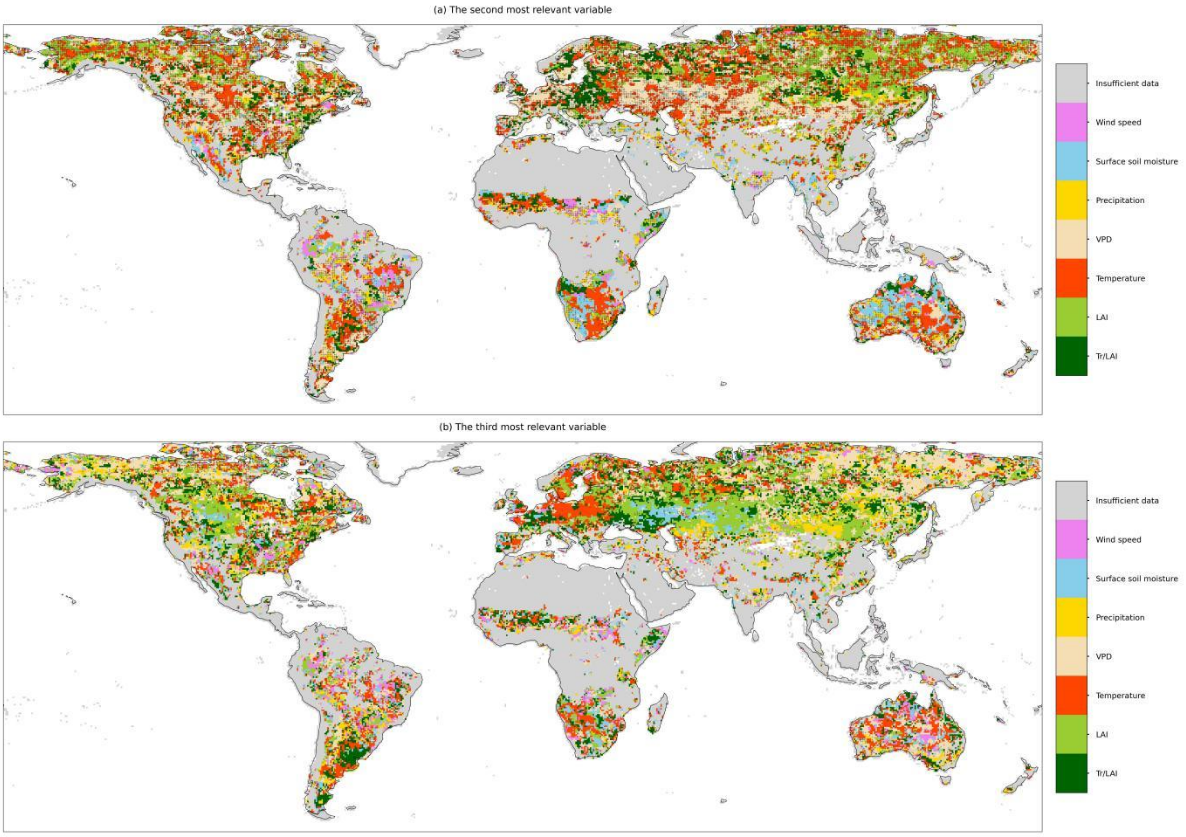

Figure S2. Spatial distribution of the second and third most important variables in regulating SM–VPD coupling. Symbols "+" denote that the ratio between the second (or the third) variable and the first variable is greater than 0.8.

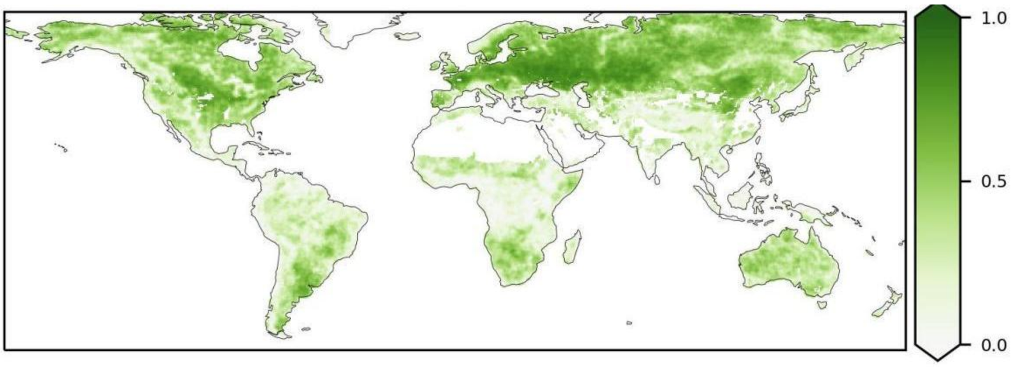

Figure S3: Random forest model performance represented by out-of-bag R^2 using absolute values of predictors.

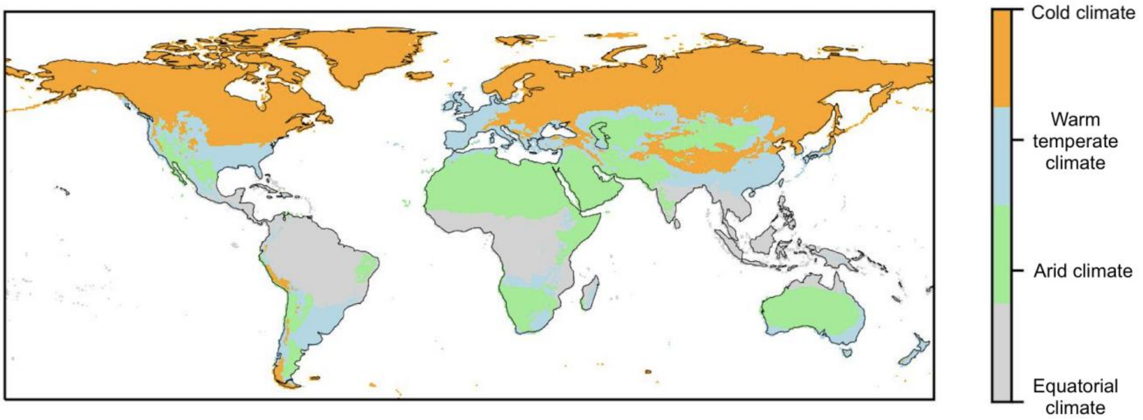

Figure S4: Geographic distribution of four main types of climates.

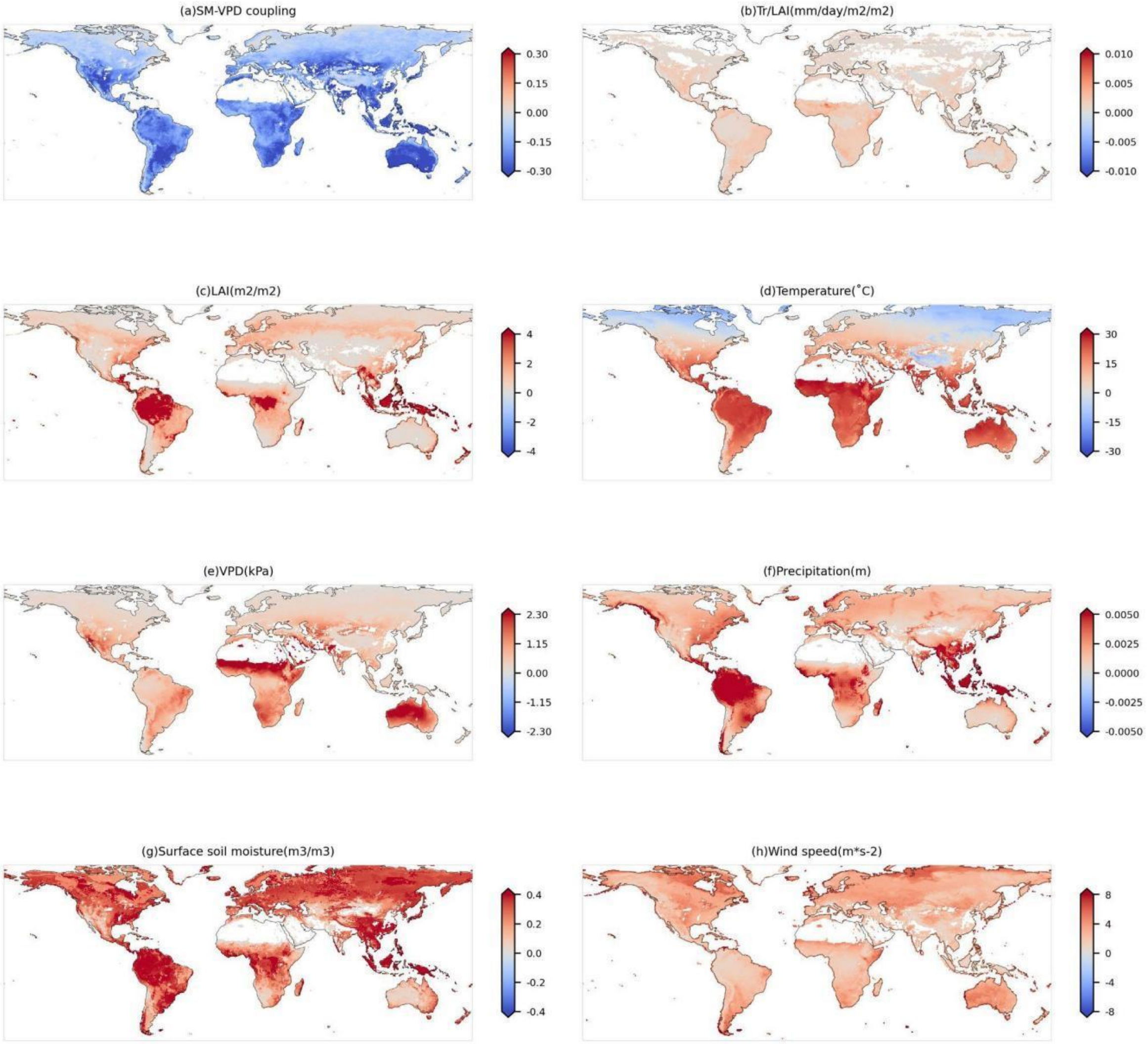

Figure S5. Averages of SM–VPD coupling and averages of its potential drivers.

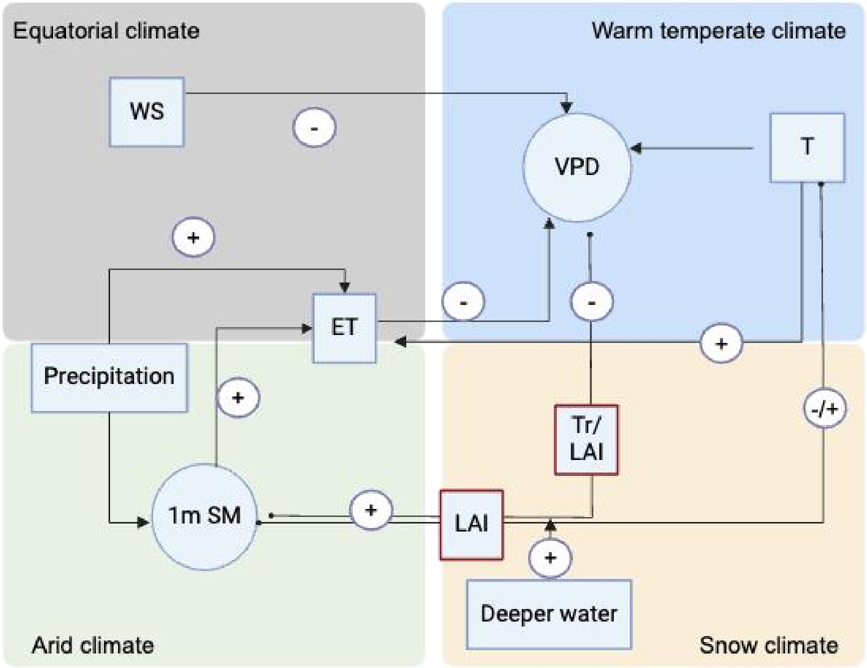

Figure S6: A schematic illustrating the potential impact pathways of vegetation structure and physiology (in red) and the hydro-meteorological variables (in blue) on SM–VPD coupling across climates. Background climates highlight the underlying main influential variables presented in Figure 3. WS: wind speed, VPD: vapour pressure deficit, T: temperature, SM: soil moisture, ET: evaporation, LAI: leaf area index, Tr/LAI: Transpiration per leaf area. +/- indicate potential physical links but note that they may differ across extreme dry or wet ecosystems.

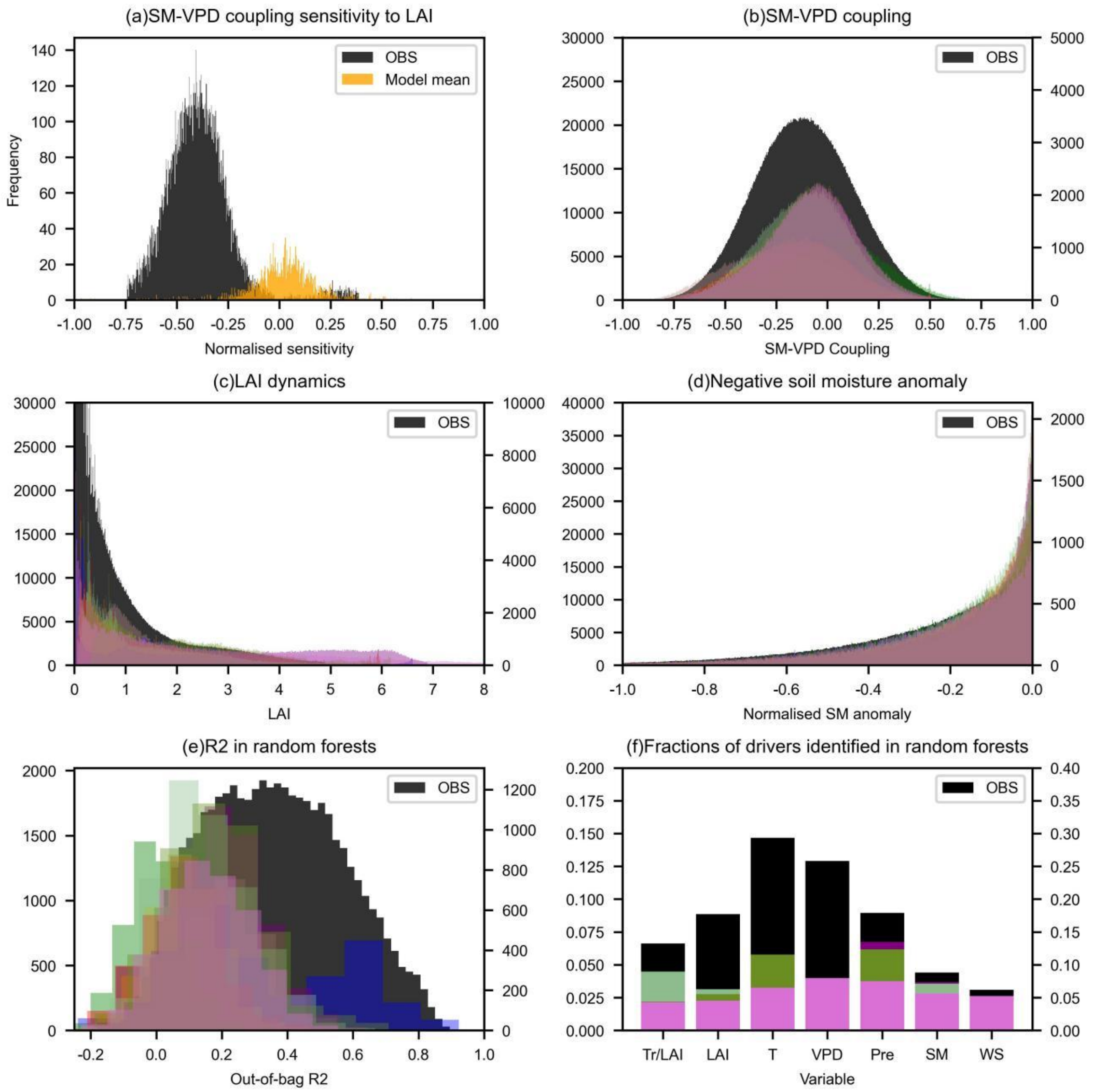

Figure S7: The distribution of SM–VPD coupling sensitivity to LAI and some relevant variables from observations (OBS) (black color) and Earth system models (Model, other color). (a) Normalised coupling sensitivity. (b) The coupling between SM and VPD calculated by partial correlation. (c) Absolute LAI variations. (d) Normalised SM anomaly as ERA5-Land SM and SM from Earth system models have different units. (e) Out-of-bag R^2 when training random forest for each grid cell to investigate variable importance and infer variable sensitivity. (f) Counting variable importance by considering three most important variables from SHAP importance results. WS: wind speed, VPD: vapour pressure deficit, Pre: precipitation, T: temperature, SM: soil moisture, ET: evaporation, LAI: leaf area index, Tr/LAI: Transpiration per leaf area. Observation-based results are in black color, averages of ESMs are in orange in (a), and individual ESMs are in other colors.

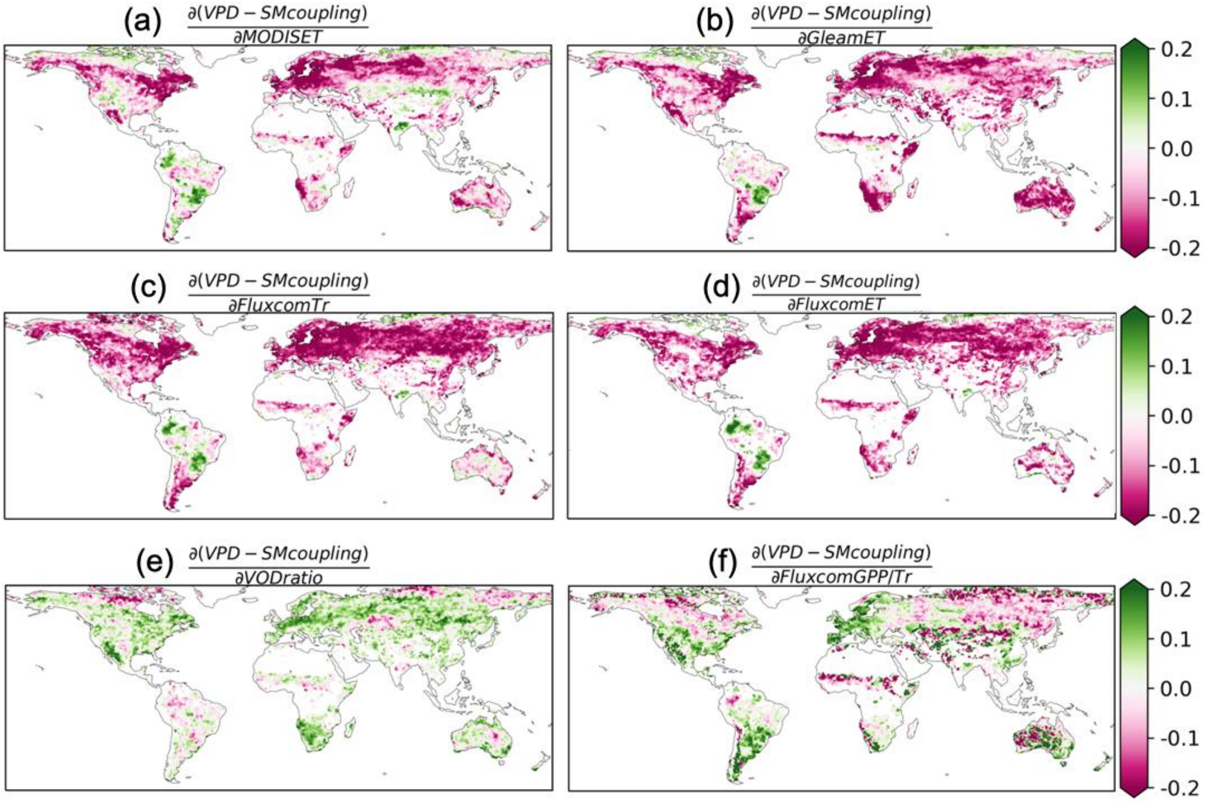

Figure S8: The sensitivity of SM–VPD coupling to different observation-based vegetation products. GPP: Gross primary productivity; Tr: Transpiration; ET: Evapotranspiration; VOD: Vegetation optical depth.

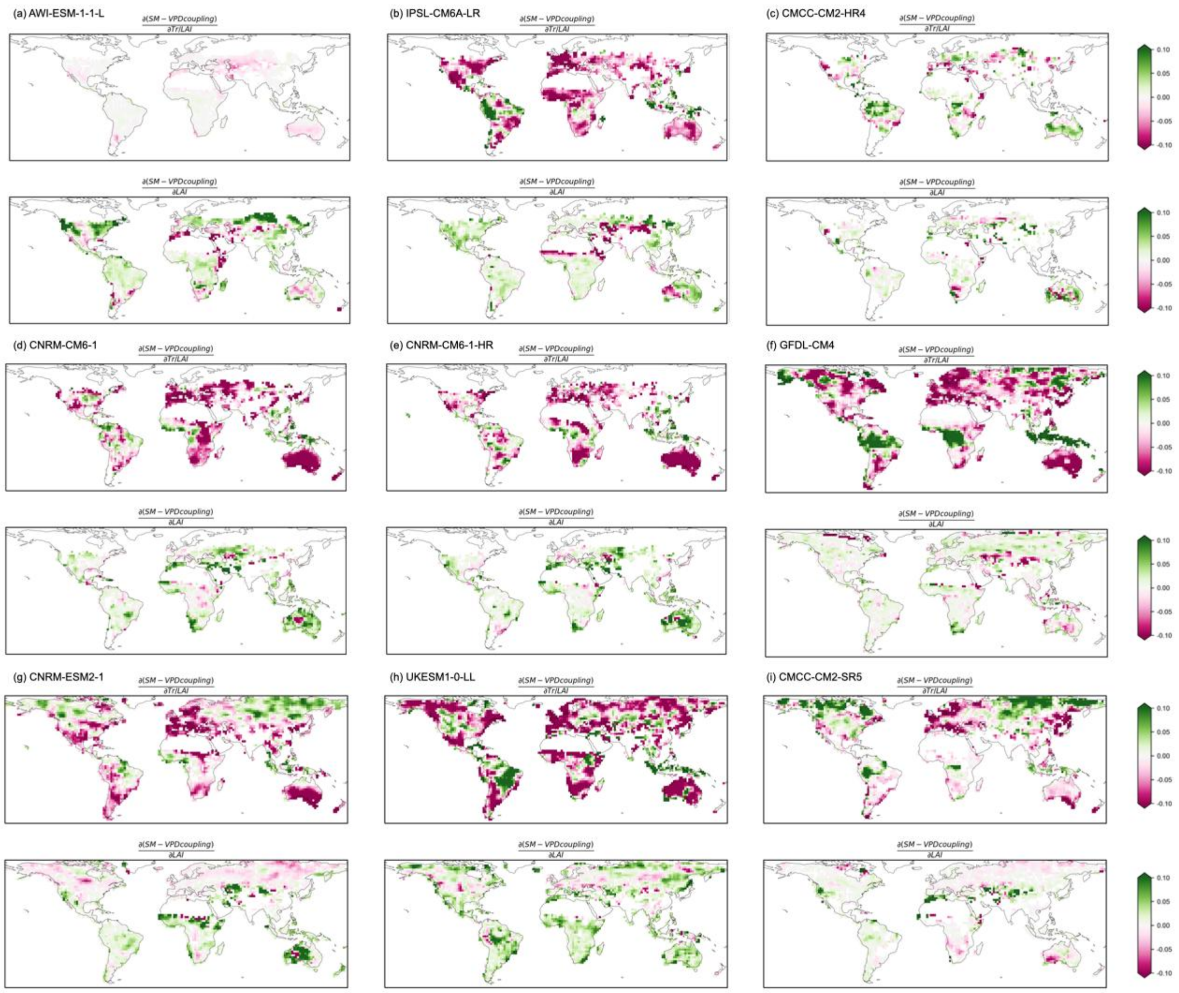

Figure S9: The sensitivity of SM–VPD coupling to vegetation physiology and structure from different Earth System Models.

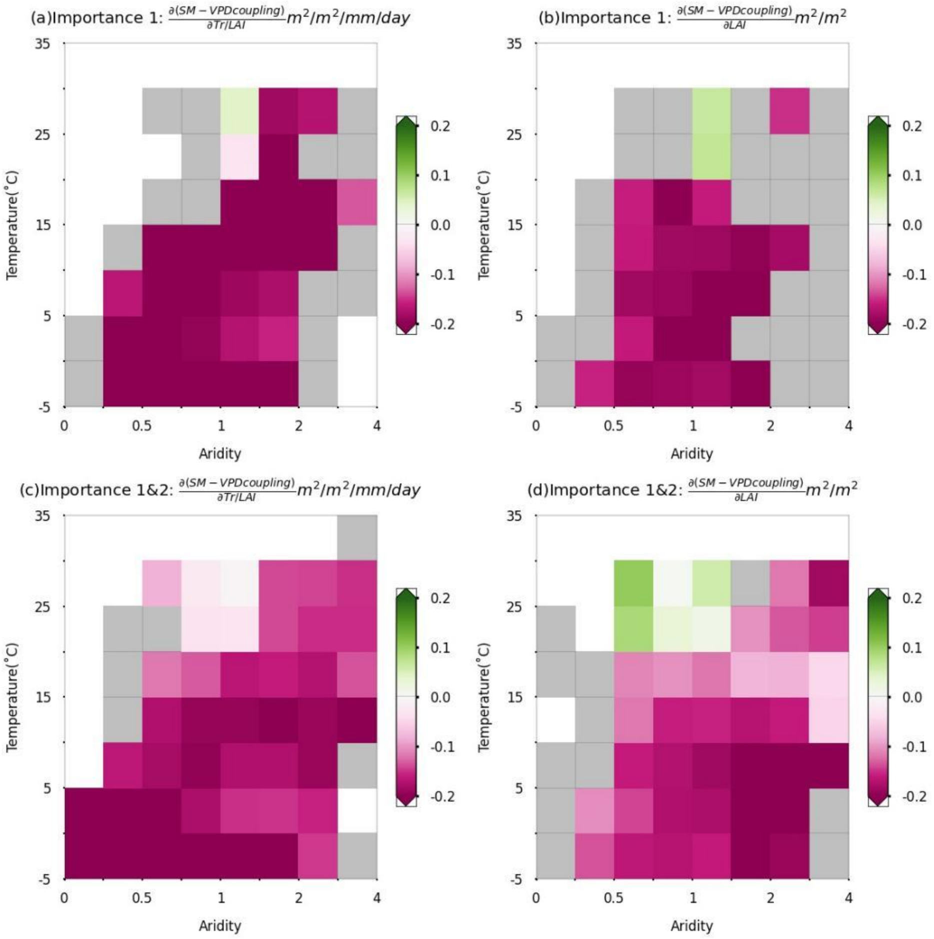

Figure S10: Similar to observation-based results in Figure 5 but removing regions that ET or LAI are not (a-b) a first-order or (c-d) a first- and second-order influential variables of SM–VPD coupling.

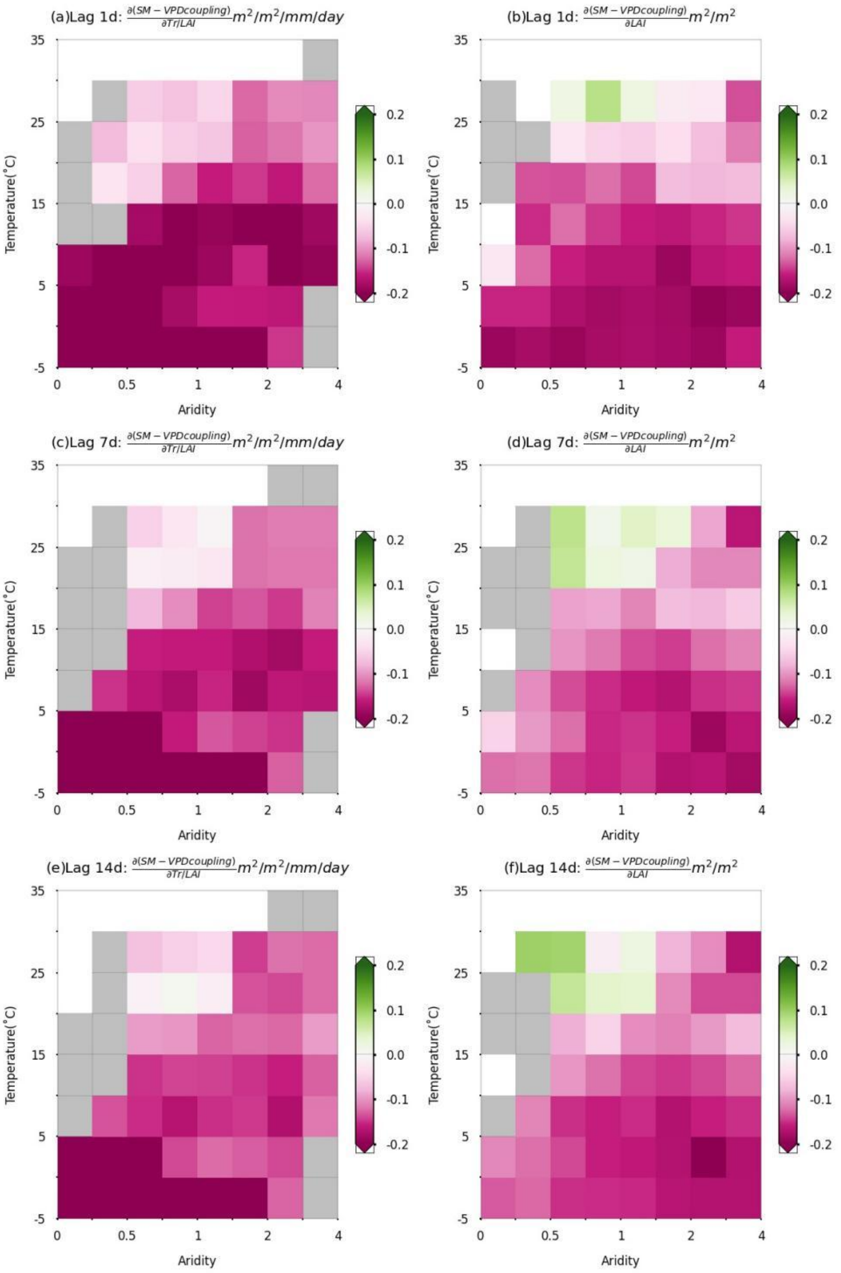

Figure S11: Similar to Figure 5, but SM–VPD coupling is calculated by the partial correlation between (a-b) 1-day lagged SM and concurrent VPD by controlling 1-day lagged VPD, (c-d) 7-day lag, and (e-f) 14-day lag.

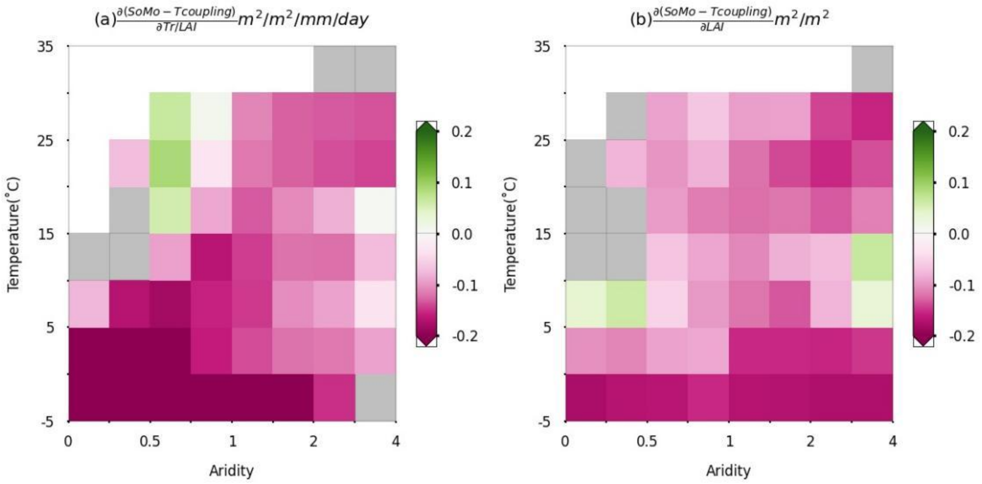

Figure S12: Similar to Figure 5 but replacing ERA5-Land SM by SoMo.ml SM, and replacing ERA5-Land VPD by temperature.

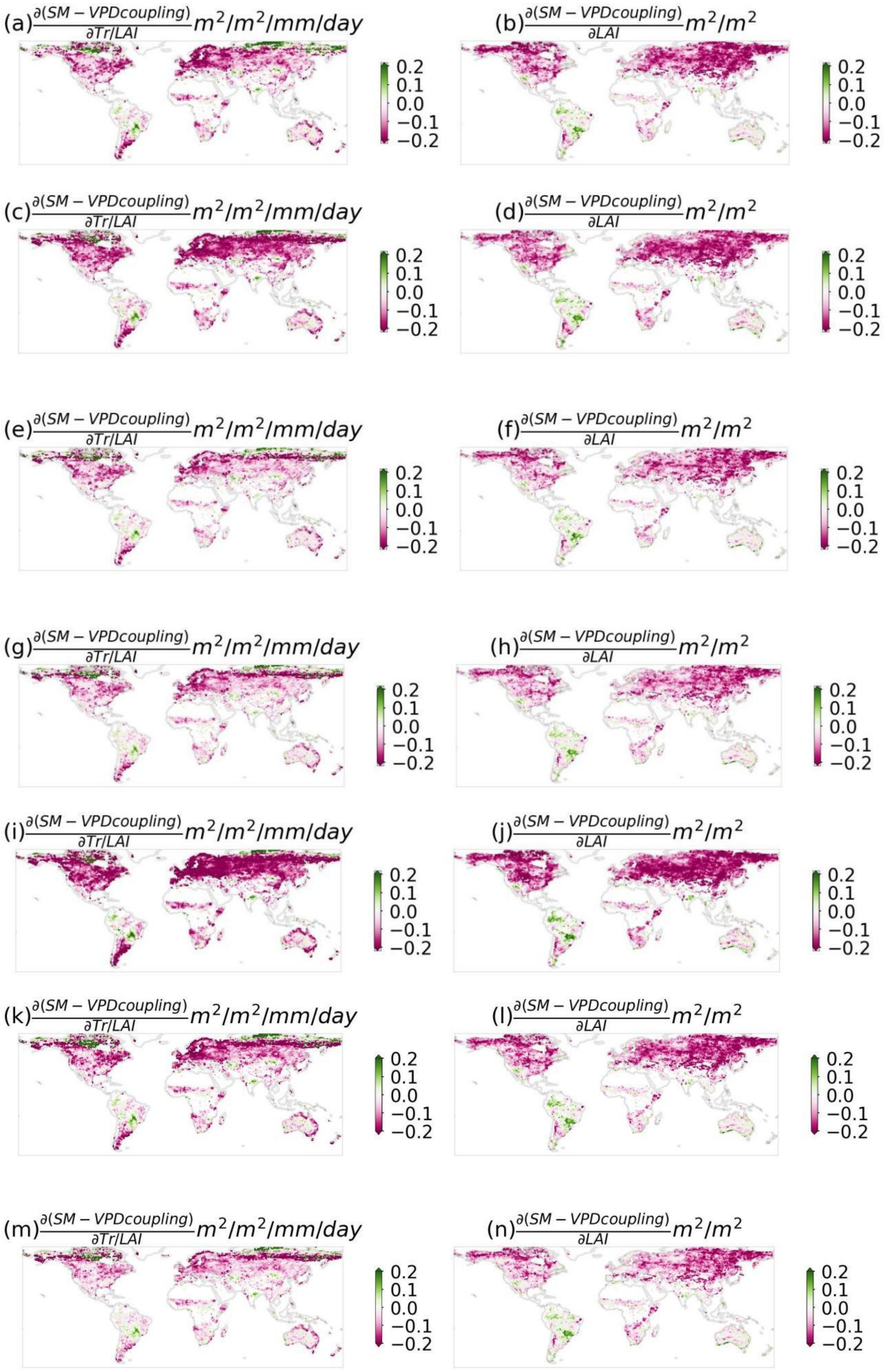

(a) $\frac{\partial(SM - VPDcoupling)}{\partial Tr/LAI} m^2/m^2/mm/day$
0.2
0.1
0.0
−0.1
−0.2
(b) $\frac{\partial(SM - VPDcoupling)}{\partial LAI} m^2/m^2$
0.2
0.1
0.0
−0.1
−0.2
(c) $\frac{\partial(SM - VPDcoupling)}{\partial Tr/LAI} m^2/m^2/mm/day$
0.2
0.1
0.0
−0.1
−0.2
(d) $\frac{\partial(SM - VPDcoupling)}{\partial LAI} m^2/m^2$
0.2
0.1
0.0
−0.1
−0.2
(e) $\frac{\partial(SM - VPDcoupling)}{\partial Tr/LAI} m^2/m^2/mm/day$
0.2
0.1
0.0
−0.1
−0.2
(f) $\frac{\partial(SM - VPDcoupling)}{\partial LAI} m^2/m^2$
0.2
0.1
0.0
−0.1
−0.2
(g) $\frac{\partial(SM - VPDcoupling)}{\partial Tr/LAI} m^2/m^2/mm/day$
0.2
0.1
0.0
−0.1
−0.2
(h) $\frac{\partial(SM - VPDcoupling)}{\partial LAI} m^2/m^2$
0.2
0.1
0.0
−0.1
−0.2
(i) $\frac{\partial(SM - VPDcoupling)}{\partial Tr/LAI} m^2/m^2/mm/day$
0.2
0.1
0.0
−0.1
−0.2
(j) $\frac{\partial(SM - VPDcoupling)}{\partial LAI} m^2/m^2$
0.2
0.1
0.0
−0.1
−0.2
(k) $\frac{\partial(SM - VPDcoupling)}{\partial Tr/LAI} m^2/m^2/mm/day$
0.2
0.1
0.0
−0.1
−0.2
(l) $\frac{\partial(SM - VPDcoupling)}{\partial LAI} m^2/m^2$
0.2
0.1
0.0
−0.1
−0.2
(m) $\frac{\partial(SM - VPDcoupling)}{\partial Tr/LAI} m^2/m^2/mm/day$
0.2
0.1
0.0
−0.1
−0.2
(n) $\frac{\partial(SM - VPDcoupling)}{\partial LAI} m^2/m^2$
0.2
0.1
0.0
−0.1
−0.2

Figure S13: Similar to Figure 4 but (a-b) present results of SM-VPD coupling sensitivity to normalised transpiration and LAI where the growing season for each grid cell is determined by the temperature greater than 5˚C and gross primary productivity of vegetation greater than 0. (c-d) LAI (or ET) is removed when quantifying the sensitivity to ET (or LAI) in random forests, to test if the high correlation between ET and LAI strongly affects the sensitivity quantification. (e-f) present observation-based results from 2003-2014. (g-h) present results when seasonality is included as one of the random forest predictors. (i-j) present results when surface SM and VPD are not included in random forest models. (k-l) present results when the parameter of the LOWESS filter is changed from 0.4 to 0.3, and (m-n) present results when incoming shortwave radiation is included in random forest models.

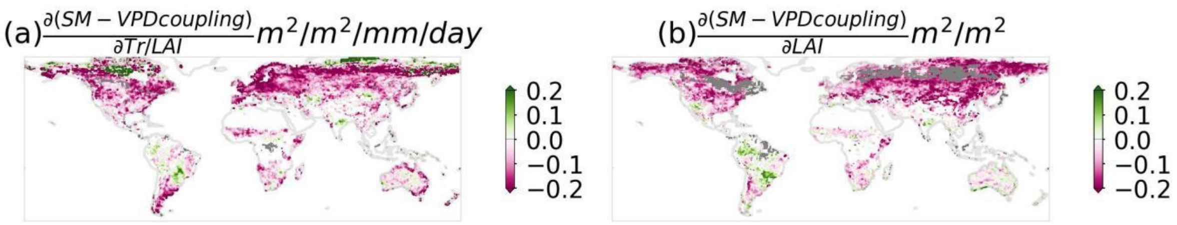

Figure S14: Similar to Figure 4, but with regions masked (grey) where strong multi-collinearity is present, indicated by a variance inflation factor greater than 10.

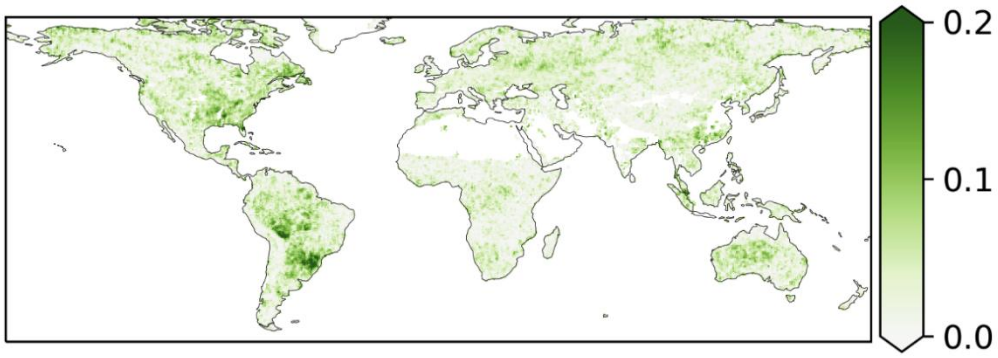

Figure S15: The difference of random forest model performance with and without including surface SM and VPD as predictors. The performance is represented by out-of-bag R^2.

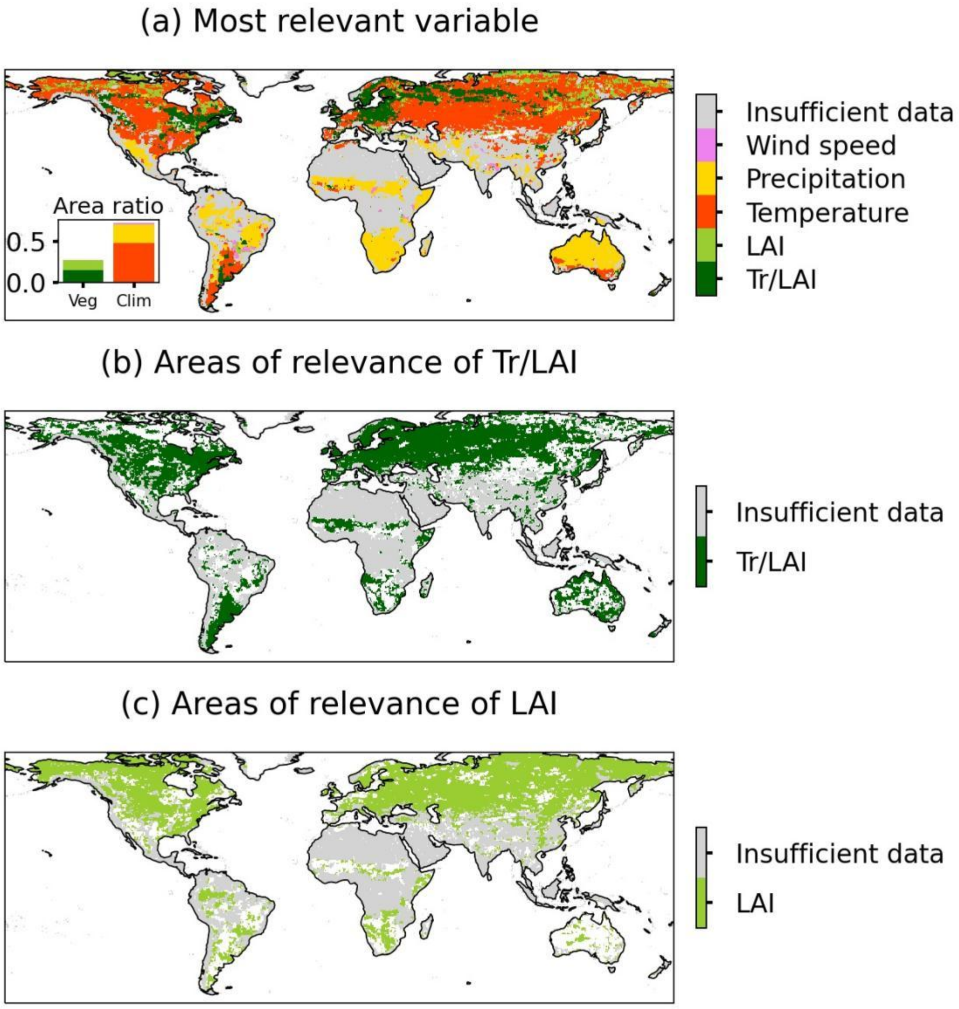

Figure S16: Similar to Figure 2 (b-d) but for variable relevance without including surface SM and VPD as predictors in random forest models.
